# Deficit in attentional capture by social-value related conditioned stimuli — a new perspective to understand the social-reward learning disorders among individuals with depressive symptoms

**DOI:** 10.3389/fpsyt.2026.1692037

**Published:** 2026-02-04

**Authors:** Jinsheng Hu, Xiaoning Zhao

**Affiliations:** Department of Psychology, Liaoning Normal University, Dalian, China

**Keywords:** attentional capture, classical conditioning, depressive symptoms, oculomotor, reward learning, social value

## Abstract

**Objectives:**

This study investigated social-reward learning disorders in individuals with depressive symptoms by examining attentional resources to be captured by social-value related conditioned stimuli.

**Methods:**

We utilized a modified additional singleton paradigm in which the prominent distractor was characterized by a stimuli associated with large- or small- social value. In Study 1, 49 participants with minimal depressive symptoms(BDI-II *≤* 13 & dimension of depressive symptoms on the SCL-90-R *≤* 2.10) and 49 with obvious depressive symptoms(BDI-II *≥* 14 & dimension of depressive symptoms on the SCL-90-R > 2.10) were included in the control and depressed groups, respectively. They were asked to find the response target and make a judgment related to the target by conducting a keypress response. In Study 2, 55 participants with obvious depressive symptoms and 54 with minimal depressive symptoms were asked to fixate on the target as quickly as possible.

**Results:**

In the keypress response task, regarding the influence on response time, the interference effect of large-value distractors in the depressed group was significantly less than that in the control group. In the fixation-response task, compared with the control group, participants of the depressed group also showed less bias of oculomotor capture by social-value distractors.

**Conclusions:**

For individuals with depressive symptoms, insufficient interference of social-value distractors was revealed both in the manual response and eye-tracking tasks. Those with depressive symptoms displayed deficits in attentional resources to be captured by social-value related stimuli.

## 1 Introduction

Social pleasure is important for relieving pressure from stressful work and study environments. Socially anhedonic individuals often feel less pleasant about social interactions, gradually withdraw from them, and develop deficient social functions. With poor social performance, individuals with social anhedonia may receive more negative social feedback, which aggravates social withdrawal and subsequent emotional disorders. The Diagnostic and Statistical Manual of Mental Disorders (DSM-5) identifies anhedonia as a key clinical feature of major depressive disorder (1). Social anhedonia, which refers to experiencing less social pleasure from benign interpersonal interactions, such as approval from colleagues or superiors and help from a working assistant, is common among individuals with depressive symptoms, and gradually received attention from theoretical researchers and clinical therapists (2–6). Reward processing was dissected into three separable psychological components by the researchers: “likes (hedonic impact),” “wants (incentive salience),” and “learning (predictive associations and cognitions)” (7). With regard to social rewards, individuals with depression displayed abnormalities in all three psychological components. Regarding “learning,” individuals with depression showed a reduced capacity to adjust future behavior based on social positive feedback (8, 9).

Recent researches have concentrated on investigating reward-learning disorders in individuals with depression through the lens of attention (10). According to the associative-learning attention model, attentional bias toward associated stimuli is an important indicator of association acquisition (11, 12). Reward-related conditioned stimuli have a clear advantage in capturing attention; however, this advantage is not evident in individuals with depression (10). Behavioral studies indicate that conditioned stimuli linked to scores/money fail to affect the response time for individuals with depressive symptoms (13–15). In a study using eye-tracking techniques, individuals with depression also lacked gaze preference for auditory-enjoyment-related stimuli (16). Although eye-tracking have been employed to explore how reward-associated stimuli capture attention among individuals with depression, researches have largely overlooked social rewards, the most common form of rewards in daily life.

For the processing of social reward, from plenty of evidences for dysfunctional neural networks and abnormal electrophysiological activities, in the phase of reward anticipation, researches demonstrated that individuals with depression lacked the motivation to approach social rewards and exhibited fewer expectations of positive social feedback (17–20); in the phase of reward consumption, the initial binary evaluation and processing salience to social rewards were also proved to be blunt for individuals with depression (21–25). Researches had shown that the responsiveness to social rewards was an important mediating factor for the early life interpersonal stress and the occurrence of depression (26, 27). Severe social and interpersonal pressure during early life, especially during adolescence, will gradually reduced the responsiveness to social rewards, eventually making individuals develop depressive symptoms. Besides, the abnormal processing of social rewards is a hereditary symptom of depression that should not be overlooked. Apart from patients with depression themselves, the offspring of them also have a lower sensitivity to social rewards such as social acceptance (28). Moreover, from the perspective of intervention, enhancing sensitivity to social rewards is a crucial step for cognitive and behavioral therapy strengthening social connection and improving symptoms related to depression and anxiety (19). It can be seen that the abnormal processing of social rewards in people with depression has been widely confirmed, and the related researches hold significant value for the depressive symptom prediction and intervention. Regarding investigating the reward learning disorder of the depressed population, focusing on the attentional processing toward social-reward cues for individuals with depressive symptoms is also worthy of the attention.

Exploration for attentional processing toward value-related stimuli has been conducted within both operant and classical conditioning frameworks. In both frameworks, reward-related conditioned stimuli all have an advantage in attentional capture (29, 30). The researchers have concentrated on the operant conditioning framework to reveal the characteristics of attentional capture by reward-related conditioned stimuli among individuals with depression. In these studies, during the conditioning phase, the acquisition of rewards was based on the correct and quick responses to the specific target. Thus, the target gradually evolves into a reward-related conditioned stimulus. At the test task, the attentional bias toward conditioned stimuli was more like an “attentional habit” to gain rewards. In addition to this, carrying out the related work under the classical conditioning is also valuable, as the conditioned stimuli under the classical conditioning hold psychological salience by possessing signal value rather than the response value of the operant conditioning. The work under the classical conditioning framework can enhance our understanding of atypical attentional processing to reward-associated conditioned stimuli among individuals with depressive symptoms.

In consideration of the aforementioned points, our study aimed to investigate the phenomenon of attentional capture by stimuli linked to social rewards during the process of classical conditioning among individuals with depressive symptoms. We employed the additional singleton paradigm, a highly effective method for studying attentional capture. In this task, the interference effect of a singleton stimulus on executive-control networks demonstrates attentional capture by the singleton stimulus (31). For our study, social reward associated stimuli were set as the singleton stimuli, one kind of singleton stimuli as the large-social reward associated stimuli and the other kind as the small-social reward associated stimuli. The enhanced interference of large-value distractors compared to the small-value distractors reflected the degree of attentional capture by the distractors associated to social value. In order to provide a more comprehensive understanding for the attention-capturing characteristics of social-value distractors, two studies were conducted. In these studies, we separately investigated the interference effect of social-value distractors in two behavioral execution system, namely, manual keypress and fixation-response.

Among individuals with depressive symptoms, reduced social reward-learning ability reflects their poor performance in social-reward association acquisition. Thus, we hypothesized that fewer attentional resources would be captured by social-value stimuli among individuals with depressive symptoms, based on the associative learning attention model. They would exhibited less disruption from large-value distractors in both the keypress task (Study 1) and the fixation task (Study 2) compared with the healthy controls. Regarding overt attention, fewer oculomotor captures by large-value distractors would also be observed among individuals with depressive symptoms than healthy individuals.

Furthermore, as a supplementary investigation, our study attempted to infer the mechanisms for attentional resources to be captured by social-value distractors from the aspect of visual suppression processing. The visual system was believed to employ two distinct mechanisms to suppress the attentional capture by the distractors: reactive suppression and proactive suppression (32, 33). Reactive suppression emphasized the process in which attention rapidly detours from a stimulus after being captured by it. Proactive suppression referred to the decline in the attentional priority of the distracting stimulus before the stimulus was presented. From the very beginning, attention would not be firstly allocated to the distractor. Researchers had differentiated the two mechanisms by exploring the time course of oculomotor capture by the distractor. On one hand, since reactive suppression occurred after the stimulus was presented, its impact should be manifested in the long saccade latency resulting from the attention transition before being captured by the interfering stimulus (34, 35). However, when the suppression failed, a clear saccade toward the distractor would be emitted. On the other hand, the intensity of proactive suppression depended on the degree of saccadic advantage that the stimulus representation occupied on the early attentional priority map (36, 37). If the stimulus had a high priority of attentional allocation, it would fastly attract the gaze, and from another perspective this reflected that the effect of proactive suppression was reduced. A short saccade latency for the distractor was accompanied by the non-prominent proactive suppression.

For the time course of oculomotor capture by social-value distractors, on condition that the oculomotor capture bias for large-value distractors occurred more frequently when the latency of the saccade was short, it was proved that proactive suppression was not obvious, the social-value stimuli had a high attentional priority and a clear advantage in oculomotor capture; when the capture bias for large-value distractors occurred more frequently when the latency of the saccade was long, this reflected that reactive suppression was aroused but mostly failed, thereby leading to saccades directed toward the distractor.

## 2 Study 1

### 2.1 Method

#### 2.1.1 Participants

A power analysis using G*Power 3.1.9.4 (38) was conducted to determine the number of participants before the study. We referred to a research which reported the difference in emotional expression recognition between individuals with high anxiety and those with low anxiety based on an effect size of Cohen’s *d =* 0.76 (39). As revealing the emotional cognitive abnormality for people with affective disorders was also our focus, we adopted this effect size to detect the difference between the healthy controls and individuals with depressive symptoms on attentional bias to social-value related stimuli. The results of power analysis showed that a group size of 38 was enough to achieve a power of 0.90 and α=0.05.

Participants were drawn from a mental health survey for undergraduate students. Prior research indicated that a Beck Depression Inventory-Second Edition (BDI-II) (40) score exceeding 13 signified the presence of depressive symptoms (41, 42). The latest norms for the Chinese Symptom Checklist-90-Revised (SCL-90-R) (43) reported a mean of 1.52 with a standard deviation of 0.58 for depressive symptoms (44). Consequently, following a comprehensive analysis of G-Power and an evaluation of the factors that led to the exclusion of participants, a sample of 55 students was initially selected to form the depressed group on the basis of a BDI-II score *≥* 14 and a score for the dimension of depressive symptoms on the SCL-90-R > 2.10 in the mental health screening, which was more than the number that we had planned in the pre-registration (https://osf.io/65yrt).

They were contacted through a reserved phone number and invited to join in the study. In the end, 52 students agreed to participate. Meanwhile, 52 students with a BDI-II score *≤* 13 and the scores for all symptom dimensions in SCL-90-R within the normal range were also successfully invited to form the control group. Healthy controls all had negative overall characteristic of the symptoms. Prior to experiment, participants underwent a structured clinical interview based on the DSM-IV-TR Axis I Disorders, Research Version, Non-Patient Edition (SCID-I/NP) (45). Depressed participants with any Lifetime Axis I disorders other than depression were excluded from the depressed group, and healthy controls with any lifetime Axis I disorders were excluded from the control group. For all participants, the exclusion criteria also included: (1) seizure disorder, (2) drug dependence, (3) use of psychotropic drugs, and (4) a history of mental illness or neurological disease. Due to insufficient valid data, for the data analysis, the exclusion of three participants belonging to the depressed group as well as three healthy controls was necessary. Final control group consisted of 49 participants, while the depression group also included 49 participants.

The two groups exhibited no substantial disparities with respect to sex distribution or age. It was reported by all participants that their vision was either normal or had been corrected to normal. As indicated by the BDI-II scores on the same day of the experiment, the majority of depressed participants exhibited moderate(BDI-II: 20–29) and severe (BDI-II: *≥* 30) depressive symptoms, seven showed mild depressive symptoms (BDI-II: 14–19). The control group comprised participants with minimal level of depressive symptoms (BDI-II: *≤* 13). On the experiment day, participants also completed the Behavioral Inhibition System/Behavioral Activation System(BIS/BAS) scale (46). Compared with healthy controls, depressed participants exhibited notably diminished incentive to seek reward and novelty and decreased sensitivity to reward. Besides, depressed participants also had significantly higher punishment sensitivity than the healthy controls. All experiments reported in this article were conducted in accordance with the Declaration of Helsinki and received approvals from our institution’s research ethics committee. Before the experiment began, each participant signed a written informed consent form and was paid after completing the experiment. **Table 1** (left column) outlined the key characteristics for both the depressed and control groups.

**Table 1:** Demographic and psychopathological profiles of two groups in Studies 1 and 2.

		Study 1			Study 2	
Participants' profiles	Control (n=49)	Depressed (n=49)	Statistics	Control (n=55)	Depressed (n=54)	Statistics
Demographics, M (S.D.)						
Age	20.9(1.9), 18-24	20.0(2.5), 17-27	*t* = 1.8, *p =* 0.078	21.0(1.9), 17-26	20.6(2.7), 18-35	*t* = 0.8, *p =* 0.411
Gender, male(female)	23(26)	24(25)	*χ^2^<*0.1, *p* = 0.840	26(29)	25(29)	*χ^2^<*0.1, *p* = 0.919
Handedness, right/left	49/0	49/0	N/A	55/0	54/0	N/A
Psychopathological features (on the same day of the experiment), M (S.D.), range (min-max)						
BDI-II	6.0(4.3), 0-13	25.7(5.3), 14-33	*t* = - 20.2, *p* < 0.001***	3.8(4.0), 0-13	22.4(7.8), 14-40	*t* = - 15.6, *p* < 0.001***
SCL-90-R-DEP	1.2(0.1), 1.0-1.5	2.8(0.5), 2.2-4.2	*t* = - 20.8, *p* < 0.001***	1.2(0.2), 1.0-1.7	2.9(0.5), 2.2-4.2	*t* = - 21.4, *p* < 0.001***
SCL-90-R-ANX	1.2(0.2), 1.0-1.6	2.5(0.7), 1.2-4.6	*t* = - 13.3, *p* < 0.001***	1.1(0.2), 1.0-1.6	2.6(0.6), 1.6-4.5	*t* = - 16.1, *p* < 0.001***
BIS	20.0(3.4), 14-28	21.7(3.1), 16-28	*t* = - 2.4, *p* = 0.017*	19.6(3.8), 9-28	21.9(3.8), 9-28	*t* = -3.2, *p* = 0.002**
BAS _Total_	41.2(4.8), 32-50	39.2(4.0), 29-51	*t* = 2.3, *p* = 0.023*	40.2(3.7), 31-48	39.3(3.6), 33-49	*t* = 1.2, *p =* 0.219
BAS _RR_	17.5(2.0), 14-20	16.3(1.9), 12-20	*t* = 3.0, *p* = 0.003**	17.0(1.9), 14-20	16.3(1.7), 12-19	*t* = 2.1, *p* = 0.038*
BAS _D_	12.3(2.0), 8-16	11.4(1.7), 8-15	*t* = 2.3, *p* = 0.023*	12.0(1.7), 7-16	11.2(2.0), 7-16	*t* = 2.2, *p* = 0.029*
BAS _FS_	12.1(1.9), 8-16	11.3(1.8), 8-15	*t* = 2.0, *p* = 0.044*	12.2(1.8), 10-16	11.5(1.6), 9-14	*t* = 2.4, *p* = 0.020*

BDI-II, Beck Depression Inventory-Second Edition; SCL-90-R-DEP, depression dimension of the Symptom Checklist-90-Revised; SCL-90-R-ANX, anxiety dimension of the Symptom Checklist-90-Revised; BIS, Behavioral Inhibition System Scale; BAS _Total_, full scale of the Behavioral Activation System Scale; BAS _RR_, BAS _Reward Responsiveness_, subscale of the Behavioral Activation System Scale; BAS _D_, BAS _Drive_, subscale of the Behavioral Activation System Scale; BAS _FS_, BAS _Fun Seeking_, subscale of the Behavioral Activation System Scale. *p 0.05, **p 0.01, ***p<0.001.

#### 2.1.2 Experimental apparatus

A 19-inch LCD screen with a resolution of 1920×1080 and a rate of refresh of 60H_Z_ was used to display the stimuli. Participants were asked to perform their response on a standard keyboard. E-prime2.0 was used to present the experimental procedure and acquire data.

#### 2.1.3 Stimuli presentation

All trials comprised three distinct stages:taking a fixation to the screen, responding to the target in the search display and receiving the behavior feedback. Images for each stage were created on a background colored black (RGB: 0,0,0) by employing Adobe Photoshop. A white cross (viewing angle: 0.6°×0.6°) appeared at screen’s center during the fixation phase. In the response stage, there were five circles and one diamond evenly spaced around the fixation point on a fictional circle with a diameter of 10.2°. The circles all had a diameter of 2.3°, while the diamond’s side measured 2.1°. In the majority of trials, four circles and the diamond had gray (RGB: 127,127,127) borders, while the remaining one circle was featured with a blue(RGB:0, 133, 254) or orange(RGB:254,149,0) border. A line which featured a visual angle of 1.43° and colored in white (RGB:245,247,249) was inside each shape. The line of the circle was inclined at 45° to either the clockwise or counterclockwise direction in random, while the diamond’s line was oriented either vertically or horizontally. The response feedback was displayed at the center of the screen, which consisted of a 2.0°×2.0° light grey (RGB:169,163,191) rectangle, images of a smiling and peaceful expression of the same model, and notifications for timeout and judgement errors. The faces’ feedbacks both had the same size, color, and location as the rectangle. The faces were from ten female and the same number male models taken from AR face database (47). In each group, the presentation frequency of each model was balanced.

#### 2.1.4 Design

The smiling and peaceful facial expressions were separately selected as the large- and small- social reward, as that for the study of Anderson (48). Circle borders in blue and orange separately indicated large- and small-value distractors. In each group, precisely 50% of the participants received blue as the large-value distractors and orange as the small-value distractors; the opposite was observed in the other half of the participants. The shape of diamond served as the target, with the positions of the distractor and target being randomly assigned. The task was to judge the direction for the line inside the diamond. The participants received instructions to press the ‘D’ key to make a response for horizontal direction and the ‘K’ key for vertical direction. The participants were also told that “In the first two block, if your response is correct and faster than normal level, sometimes, a smiling face expression will be presented, at other times, the peaceful face expression of the same model will be presented. In the subsequent blocks, in blocks 3 and 4, if your response is correct and faster than the 75th percentile of recorded response times(RTs) from blocks 1 and 2, in blocks 5 and 6, if your response is correct and faster than the 75th percentile of recorded RTs from blocks 3 and 4, and so on, in blocks 9 and 10, if your response is correct and faster than the 75th percentile of RTs from blocks 7 and 8, the smiling/peaceful face expression will be presented again.”

We employed a 2(group:control, depressed)*3(distractor kind:large value, small value, absent) mixed experimental design with group as the between-group factor and distractor kind as the within-group factor. A total of 400 trials were conducted, comprising 180 large-value distractor trials, 180 small-value distractor trials, and 40 distractor-absent trials. Each kind trial was uniformly allocated across 10 blocks. Trials in each block were presented randomly. For each block, the ratio that the vertical line was set inside the diamond reached 50%, and the remaining 50% was the ratio that the horizontal line was set inside the diamond. Feedback was provided at the end of each trial based on the correctness of the judgment and response time. In the first two blocks, under the premise of correct judgment, when the RT was < 800ms (based on the pre-experiment), the feedback was a smiling face expression for large-value distractor trials or a peaceful face expression for small-value distractor trials. Even under the correct premise, when RT was more than or equal to 800ms while less than 2000ms, the rectangle stimulus would be presented.

Drawing on the study conducted by Watson et al. (49), in subsequent blocks, regarding RT, the reward time-limit of the participants was determined by their own response speed. For each participant, the 75th percentile of recorded RTs from the blocks 1 and 2 was set as the reward time-limit of blocks 3 and 4, and so on, the reward time-limits of blocks 9 and 10 were the 75th percentile of recorded RTs from the blocks 7 and 8. In all blocks, for distractor-absent trials, under the correct premise, when RT was < 2000 ms, the feedback was always a rectangular stimulus. In all trials, an error prompt appeared when the judgment was wrong, and a timeout prompt appeared if RT was *≥* 2000 ms.

#### 2.1.5 Self-reported measures

Participants completed three questionnaires on the experiment day. First, BDI-II (40) measured the extent of depressive symptoms. Topics concerning death and sex were omitted for adhering to the moral standards of the experimental procedure. Total score range was 0 to 57, with a higher score indicating increasing depression. Second, BIS/BAS (46) measured the activate features of the behavioral inhibition and behavioral activation systems. The subscale of BIS assessed negative feelings toward punishment (score range: 7–28), while the BAS_Driver_ subscale evaluated motivation for reward-seeking (score range: 4–16).The BAS_Reward Responsiveness_ subscale gauged pleasure in response to rewards (score range: 5–20), and the BAS_Fun Seeking_ subscale measured the pursuit of novelty (score range: 4–16). In the above subscales, a higher score indicated a more pronounced corresponding feature. Third, SCL-90-R (43) measured current psychopathological symptoms. A 5-point Likert scale, with values from 1 (none) to 5 (severe), is utilized. For each factor, the score ranges from 1 to 5, with higher score reflecting more severe related-symptom.

#### 2.1.6 Experimental procedure

Participants were set at an acoustic room to complete the experiment. The monitor was set 60 cm from the participants. At the onset of the experiment, the participants were presented with clear instructions regarding the task to be performed and the feedback of the response. Afterward, we asked the participants to undertake a practice task comprising 20 trials with identical settings to those employed in the formal experiment, with the exception of the distractor colors (large value: red; small value: brown). Participants proceeded to the formal experiment upon achieving an accuracy of 75%.

**Figure 1:**
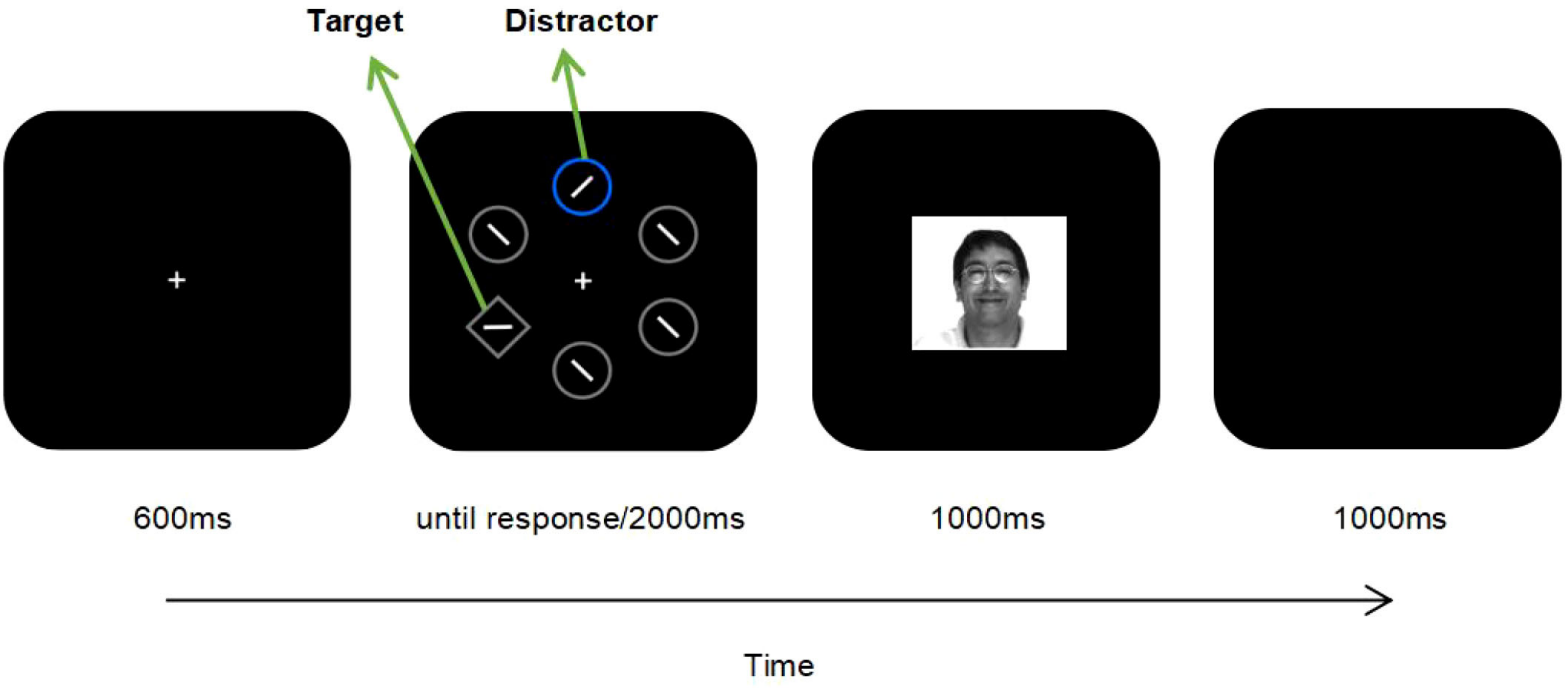
Flowchart depicting a single trial where a smiling face is achieved under the large-value distractor condition. The participant was asked to make a judgement to the direction for the line segment inside the diamond (target). Typically, one circle’s edge was colored. Colored borders (orange, blue) were large- or small-value distractors which indicated that appropriate response would obtain a smiling or peaceful face. Participants received a smiling face for a correct and quick response (RT < reward time-limit) under the condition of large-value distractor, a peaceful face for the proper response under the condition of small-value distractor, and a rectangle stimulus for the proper response when no distractor appeared. The smiling face was obtained from The AR Face Database: CVC Technical Report, 24 (1998) by Martinez A, Benavente R.

In the formal experiment, at first, the fixation point manifested itself and lasted for a period of 600 ms. Next, the stimuli were presented and participants were required to use the keyboard to respond. The stimuli disappeared only when the presentation lasted for 2000ms or the response was conducted. Response feedback was then displayed for a duration of 1000 ms. Following the disappearance of the aforementioned feedback message, the screen was blank for 1000ms as the interval of the trials. A schematic of the experimental process is shown in **Figure 1**. After completing two blocks, participants could take a break. The whole experiment lasted approximately 40–50 minutes, contingent on participants’ response state. As the pay, each participant received 15 yuan. Participants completed the self-reported questionnaires of BDI-II, BAS/BIS and SCL-90-R following the experiment.

#### 2.1.7 Statistical analyses

For the analysis of RT, trials with error judgments, timeouts, and RTs <150 ms were removed first. For each participants, trials in which RTs were more than 3SDs from the mean of each conditions were deleted. When trial-deletion rate exceeded 25%, the participant was considered invalid and all their trials were removed. Consequently, trials with three depressed participants and three healthy controls were omitted from the analysis of data. The results remained consistent even when including the previously excluded participants. Among valid participants, erroneous judgments led to the exclusion of 7.3% of trials for the control group and 7.6% for the depressed group. For the control group, 0.9% of trials were removed due to timeouts, while in the depressed group, 0.4% were removed for the same reason and an additional 0.04% were excluded because RTs were less than 150ms. Additionally, trials with extreme values, defined as RTs beyond the mean plus or minus three standard deviations, led to the exclusion of 1.2% for trials of healthy controls and 1.3% for that of depressed group.

For the analysis of accuracy, the proportion of correct response trials in which the RTs were greater than 150 ms and within three standard deviations of the mean for each experimental condition was calculated. The mean RT and accuracy for each participant’s valid trials fell within ±2.5SDs of their group’s mean. The reward interference were quantified by calculating the extent to which reaction time was prolonged (KRT _large_- KRT _small_) and the extent to which the accuracy (ACC _small_ - ACC _large_) was decreased when large-value distractors appeared than small-value distractors. The independent-samples t-test separately for KRT _large_- KRT _small_ and ACC _small_ - ACC _large_ between the two groups was used to clarify the differences in attentional resources to be captured by social-value related distractors between the depressed participants and healthy controls. The repeated-measures analysis of variances (ANOVAs) for RT and accuracy with the group (depressed, control) as the between-subjects factor and the distractor kind (large value, small value, absent) as the within-subjects factor were added to perform the further analysis. We also conducted a Hierarchical Linear Model(HLM) to continuously analyze the effect of depression on attentional bias to social-value stimuli.

### 2.2 Results

#### 2.2.1 RT and accuracy

Regarding the KRT _large_- KRT _small_, the independent-samples T test revealed that the depressed group exhibited significantly lower reward interference compared with the control group, *t*(96) = 5.08, *p <*.001, Cohen’s *d =* 1.026. Repeated-measures ANOVA was conducted to investigate the RT as a function of group (depressed, control) and distractor kind (large value, small value, absent). The distractor kind had a significant main effect, *F*(1.32, 126.29)=106.24, *p<*.001, η*_p_,^2^* = .525. The *post-hoc* test using least significant difference (LSD) indicated that when the distractor was absent, the RT was separately significantly faster than that when the large-value distractors appeared and that when the small-value distractors appeared, *ps<*.001. The RT under the presentation of large-value distractors was slower than that under the presentation of small-value distractors, *p=*.022. The group effect was not statistically significant, *F<*1.

In addition, a statistically obvious interaction of distractor kind and group was found, *F*(1.32, 126.29)=5.73, *p=*.011, η*_p_,^2^* = .056. For results of the control group, the presence of large-value distractors obviously reduced reaction speed compared to the small-value distractors, *p<*.001, whereas the results for the depressed group showed no significant difference in response time between the large- and small- value distractor conditions, *p=*.154. In both groups, the RTs when no distractor was presented were shorter than those when large- or small-value distractors were presented, *ps<*.001.

**Figure 2:**
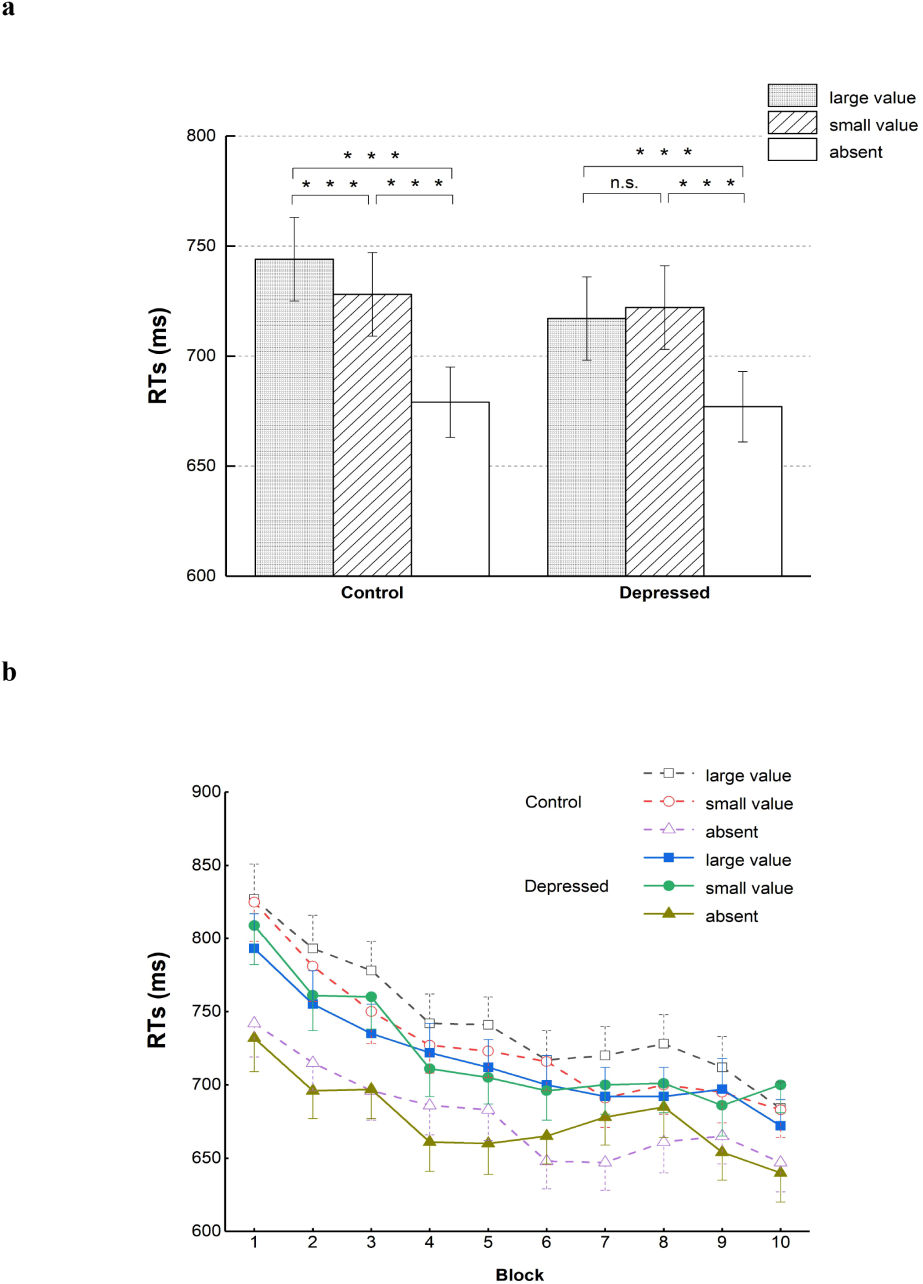
Mean RTs for both the control and depressed groups under different distractor-kind conditions **(a)** and mean RTs in ten blocks **(b)**. Error bars denote standard errors. ***p < 0.001, n.s., insignificant.

Regarding the ACC _small_ - ACC _large_, from the result of the independent-samples T test, no significant difference between the depressed group and the control group was revealed, *t*(96) = -0.19, *p =*.848. From the results of 2(group:depressed, control)×3(distractor kind: large value, small value, absent) repeated-measures ANOVA, the accuracy was significantly influenced solely by the distractor kind, *F*(1.71, 164.33)=10.11, *p<*.001, η*_p_,^2^* = .095. Neither the interaction for the two factors nor the main effect of the group was significant, distractor kind * group: *F* < 1, group: *F*(1, 96)=1.82, *p=*.181. The LSD *post-hoc* test indicated a significant decrease in accuracy when a large-value distractor or small-value distractor was present compared to its absence, large value *vs*. absent: *p<*.001, small value *vs*. absent: *p=*.007. There was no significant difference in accuracy between the large- and small-value distractor conditions, *p=*.197. See **Table 2** and **Figure 2a** for the mean RT and accuracy among different experimental conditions.

**Table 2:** Mean response times (ms) and accuracies among different distractor-kind conditions for the two groups (standard errors in parentheses, 95% confidence interval following the comma).

Indicators	Group	Large value	Small value	Absent
RT	Control	744(19), 706-781	728(19), 691-766	679(16), 646-711
	Depressed	717(19), 679-754	722(19), 685-760	677(16), 644-709
Accuracy	Control	0.91(0.01), 0.90-0.93	0.92(0.01), 0.90-0.93	0.93(0.01), 0.92-0.95
	Depressed	0.90(0.01), 0.89-0.92	0.91(0.01), 0.89-0.92	0.92(0.01), 0.91-0.94

#### 2.2.2 RTs of different blocks

An exploratory analysis was performed to conduct a comparison for interference of large-value distractors between the two groups across different blocks. A repeated-measures ANOVA with a 2×3×10 design was conducted to examine RTs, incorporating group (depressed, control) as the between-subject factor, and distractor kind(large value, small value, absent) along with block(1–10) as the within-subject factors. Consistent with the above results, the distractor kind’s main effect was significant, *F*(1.33, 127.30)=108.26, *p <*.001, η*_p_,^2^* = .530. A significant interaction of group and distractor kind was also found, *F*(1.33, 127.30)=5.58, *p =*.012, η*_p_,^2^* = .055.

In addition to these, the block’s main effect was significant, *F*(5.16, 495.79)=47.82, *p <*.001, η*_p_,^2^* = .332. The RT gradually decreased from blocks1 to 10, a trend which indicated an obvious training effect. A significant interaction of block and distractor kind was also found, *F*(12.65, 1214.14)=2.13, *p=*.011, η*_p_,^2^* = .022. Compared with the condition of distractor absent, when the distractor was presented, the declining trend of RT was more obvious. In addition, effects of group, block * group and block * group * signal type were non-significant, group: *F<*1, block * group: *F<*1, block * group * signal type: *F*(12.65, 1214.14)=1.22, *p=*.264. **Figure 2b** presents the average RTs for two groups under three distractor-kind conditions in the ten blocks.

We conducted a HLM using restricted maximum likelihood estimation to continuously verify linear effect for depression on attentional bias to social-value stimuli. RT was selected as the dependent variable, and distractor kind, BDI-II scores were selected as the predictor variables. We also controlled for the influence of anxiety levels, and set the score of anxiety dimension in the SCL-90-R as a covariate. Fixed effect for the interaction of distractor kind and the scores for BDI-II was what we focused on. The trials of large- and small-value distractor within each block were compressed into two independent observation. Each participant offered a total of 20 observations—10 for each of the large- and small-value distractor conditions. Participants was a random factor that accounted for within-subject variance. The variance inflation factors (VIFs) of the model showed no multicollinearity existed among factors (VIFs ≤ 1.46). In accordance with the study of Kreft (50), the sample size of our study (98 participants and 1,960 observations) was sufficient to calculate the interaction of cross-level variables at 90% statistical testing power. As we expected, the score of BDI-II exhibited substantial influence on the predictive relationship of distractor kind and response time, *standardized β* = 0.06(95%CI: 0.00, 0.11), *t* (159.52)=2.02, *p* = .045. The higher the score of BDI-II was, the greater of impact it had on the degree of attentional capture by social-value distractors. With the depression status as a continuous variable, this further corroborated the results of the independent-samples t-test and ANOVA. See **Table 3** for the detailed effect of each variable.

**Table 3:** HLM with RT (Experiment 1) as the dependent variable and HLM with percentage of trials involving gaze directed at distractors as the dependent variable (Experiment 2) as well as that with perc. of first saccade to the distractor as the dependent variable (Experiment 2) (Experiment 1:observations =1960, n=98; Experiment 2:observations = 1090, n=109).

Variables	RT		Perc. of gazing at distractors		Perc. of first saccade to the distractor	
	*B (95%CI)*	t	*B (95%CI)*	t	*B (95%CI)*	t
(Intercept)	735.06 (716.14,753.97)	*--*	0.15 (0.13,0.17)	*--*	0.14 (0.12,0.16)	***--***
Distractor	-0.02 (-0.07,0.04)	*t* (159.52) = -0.53	-0.07(-0.14,-0.01)*	*t* (105.53) = -2.20	-0.07 (-0.14,0.00)	*t* (95.06) = -1.98
BDI-II	-0.20 (-0.35,-0.04)*	*t* (229.94) = -2.55	0.01(-0.19,0.20)	*t* (185.45) = 0.07	0.02 (-0.19,0.22)	*t* (165.60) = 0.16
ANX	0.00 (-0.15,0.15)	*t* (199.70) = 0.02	-0.29 (-0.48,-0.11)**	*t* (99.36) = -3.14	-0.32 (-0.52,-0.13)**	*t* (99.59) = -3.32
Distractor*BDI-II	0.06 (0.00,0.11)*	*t* (159.52) = 2.02	0.07(0.00,0.13)*	*t* (105.53) = 2.00	0.06 (-0.01, 0.13)	*t* (95.06) = 1.76

Unstandardized *B* coefficients are used to present intercept effects and standardized *B* coefficients are used to present the effects of variables. Distractor: Distractor kind. The distractor kind is modeled as a discrete value, with 0 representing large-value distractors and 1 representing small-value distractors. Scales are modeled with standardized scores.*p < 0.05, **p < 0.01.

### 2.3 Discussion

The result that reflected the obvious influence of depression on attentional bias toward social-value distractors came from the analyze of RT. We found that the interference effect of high-value distractors in the depressed group was significantly lower than that in the control group. Besides, from the results of RTs in different blocks, the training effect had no influence on the difference of interference by distractors related to social value between the two groups. More importantly, based on the scores of the BDI-II scale, the level of depression was defined as a continuous variable. By establishing a hierarchical linear model, we verified the positive moderating effect of depression level on the bias of attentional capture by social-value distractors from the perspective of continuous analysis. It was reasonable to conclude that individuals with depressive symptoms exhibited a reduced pattern for attentional resources to be captured by social-value distractors.

For Study 1, under the objective to examine the case that attentional resources were captured by social-value distractors among individuals with depressive symptoms, we employed the response task relying on manual keypress to compare the impact of large-value distractors on executive-control performance between the depressed participants and healthy controls. In Study 2, following the same objective, we focused on the difference in the interference of large-value distractors between the two groups at a fixation-response task. Utilizing eye-tracking technology, we also tried to uncover the characteristics of overt capture by social-value distractors among individuals with depressive symptoms.

## 3 Study 2

### 3.1 Method

#### 3.1.1 Participants

The rules and process for the selection of participants were the same as those used in Study 1. As in the pre-registration (https://osf.io/65yrt), 112 undergraduates were selected from the same mental health survey of Study 1. Depressed and control groups comprised 56 participants each. None of them had participated in Study 1. Written informed consent was signed prior to the experiment for every participant. All participants had normal or corrected to normal vision. Participants underwent the SCID-I/NP on the experiment day. The exclusion criteria was the same as those used in Study 1. Consistent with Study 1, all participants were accepted based on the interview results. Data from one depressed participant and one healthy control were excluded due to insufficient valid data. Data from another one depressed participant were deleted for their fixation time to the target was more than 2.5 SDs later than the group mean. Data from 55 healthy controls and 54 depressed participants were included in final analysis.

The two groups did not differ significantly in age and sex distribution. From the scores of BDI-II on the experimental day, all healthy controls had minimal depressive symptoms (BDI-II: *≤* 13). The degree of depression for majority depressed participants were mild (BDI-II: 14–19) and moderate (BDI-II: 20–29), while eight depressed participants had major depressive symptoms (BDI-II: ≥ 30). As indicated by the scores of SCL-90-R, the participants in the depressed group exhibited the symptoms that exceeded the established normal range for depression toward undergraduates from China (mean = 1.52, SD = 0.58) (44). By way of contrast, the healthy controls demonstrated scores that were within the range of normal in each of the symptoms. Aggregate symptom profiles exhibited by all participants of control group manifested a negative tendency. Participants with depressive symptoms exhibited stronger negative feelings toward punishment compared to healthy controls. Participants in the depressed group showed reduced motivation for seeking reward and novelty, as well as lower receptivity to rewards compared to the control group. Refer to **Table 1** (right column) for fundamental characteristics of both groups.

#### 3.1.2 Experimental apparatus

A Dell monitor with 21-inch, operating at a refresh rate of 60 Hz, and boasting a resolution of 1024×768 pixels was used to display visual stimuli. Eye movements were recorded using a monocular Eyelink 1000 desktop-mount system, featuring a 1000 Hz temporal resolution, gaze resolution under 0.01 degrees, and gaze position accuracy of 0.5 degrees. An automatic algorithm was used to detect saccades, with a 30°/s minimum velocity and a 8,000°/s^2^ acceleration criteria. Participants used a chin rest which was positioned at a distance of 60 ms from the screen to maintain head stability. The procedure of 9-point grid calibration preceded every block.

#### 3.1.3 Stimuli presentation

Same as the Study 1, each trial comprised three distinct displays: fixation point, search array, and behavior feedback, all drawn on a black (RGB: 0,0,0) background by using Photoshop. For the fixation point, the white cross with a visual angle of 0.6°×0.6° was set at the center of the screen. The search display was identical to that in Study 1, with the exceptions: (i) shapes were all filled fully; (ii) no lines were presented within the shape. Each shape’s center was positioned at equal interval around an imaginary circle with the diameter of 7.1°. Each circle’s diameter was 2.1°, and side’s length of the diamond was 1.9°. The display of feedback remained same as in Study 1, except that there was no judgment error prompt. The models from Study 1 were selected again.

#### 3.1.4 Design

The smiling and peaceful facial expressions of the same model were respectively selected as the large- and small- social reward. As in Study 1, a 2 (group:control, depressed)*3(distractor kind: large value, small value, absent) mixed experimental design with group as the between-group factor and distractor kind as the within-group factor was also employed. The experiment consisted of five blocks with a total of 400 trials, which included 180 large-value distractor trials, 180 small-value distractor trials, and 40 distractor-absent trials. Each block was characterized by equal distribution of distractor kind and randomization of trial presentation. The signal that predicted a reward outcome was the color singleton of a circle. Participants in each group were divided such that one half received blue (RGB:0, 133, 254) circles as large-value distractors and orange (RGB:254, 149, 0) circles as small-value distractors, while the other half had the reversed color assignment. The distractor was randomly positioned in the search array, except that it was never placed close to the target.

A circular region of interest (ROI) with the diameter of 3.0° was created around the diamond, and another ROI with a larger diameter (i.e, 3.5°) was established around the color singleton. We defined the response of fixating on the target as the fixation duration in the ROI of target surpassed 100ms. After initiating the trigger of the fixation, participants received the feedback of the response. When the fixation trigger did not launch within 2000ms, “Timeout” would be displayed. In the first block, if the latency for initiating the fixation trigger was within 600ms, feedback varied by distractor kinds: a smiling face for large-value distractor trials, a peaceful face for small-value distractor trials, and a rectangular stimulus for distractor-absent trials. For each participants, similar to Study 1, for subsequent blocks, the reward time-limit was the 75th percentile of recorded RTs from the previous block. If the latency of the fixation-trigger launching was longer than the time-limit, the rectangular stimulus would be presented as the feedback.

#### 3.1.5 Experimental procedure

**Figure 3:**
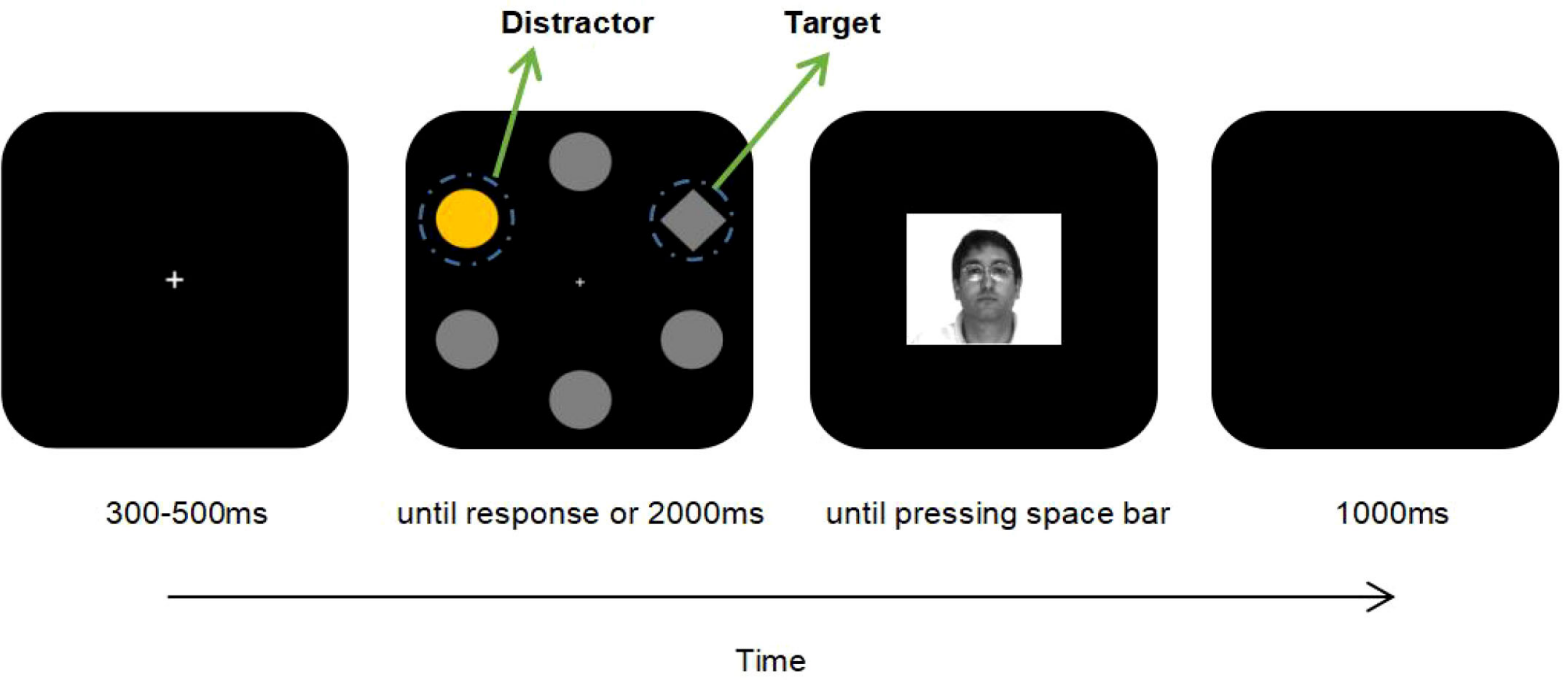
Diagram illustrating the process for a trial. The display of the peaceful face was accompanied by the appearance of a small-value distractor which was represented as an orange circle. Participants were instructed to quickly fixate on the target (i.e.,diamond) when the search display was presented. Typically, one circle was colored. The color of the distractor (orange or blue) was associated with the facial expression feedback. Participants could earn a smiling or peaceful face by responding quickly, separately under the appearance of a large- and small-value distractor. If no distractor appeared, a rectangle stimulus would be presented. ROI was delineated surrounding the target and distractor by dashed lines that were invisible to the participants. The peaceful face was obtained from The AR Face Database: CVC Technical Report, 24 (1998) by Martinez A, Benavente R.

The SR Research Experiment Builder (version 1.10.1, SR Research Ltd., Ontario, Canada) was utilized to conduct procedure. Prior to formal experiment, completion of eight practice trials with identical design to the formal trials except that the distractor had different color (i.e., green and brown) was a prerequisite for the participants. They were instructed to swiftly and precisely direct their gaze to the diamond. The instruction also included “ In the first block, if your response is faster than the normal level, as the feedback, a smiling or peaceful face expression will be presented. Otherwise, a rectangular box will be displayed. In the subsequent blocks, if your response is faster than the 75th percentile of recorded RTs of the last block, these faces will be presented again. “ The trial began once the drift correction had been successfully completed. Initially, the fixation display was presented for a duration of 300–500 ms. Following this, the search display appeared for 2000 ms or til the fixation trigger was launched. Subsequently, a feedback display appeared, which was ended by pressing the space bar. A blank screen then ensued for 1000 ms before the next trial began. Participants could take a break after completing each block. The entire experimental procedure took between 30 and 40 minutes, varying with each participant’s response time. As compensation, each participant received 20 yuan. After finishing the experiment, participants needed to fill out the same three questionnaires that even used in Study 1. Refer to **Figure 3** for the experimental procedure diagram.

#### 3.1.6 Statistical analyses

The raw eye-tracking data were exported from the EyeLink Data Viewer software(version 2.6.58 SR Research Ltd., Ontario, Canada). Trials were excluded if they involved timeouts, drift correction over 7000 ms, or first saccade latency under 80 ms. Additionally, akin to Study 1, trials for each participant where fixation time to the target exceeded 3SDs from the mean of each distractor-kind condition were excluded. As a result, all trials of one healthy control and one depressed participant were excluded due to the discarded rate exceeding 25%. Trials involving one depressed participant were deleted in that the mean fixation time to the target exceeded 2.5 standard deviations from the group mean for valid trials. It was important to note that the results of the analysis remained consistent, even when participants who had been excluded were included.

In the remaining participants’ trials, 2.6% of trials for the healthy controls along with 4.5% of trials for the depressed group were discarded because of timeout; 5.1% of trials for healthy controls and 3.5% of trials for the depressed group were excluded for the first saccade latencies were under 80ms; 1.0% of trials for the healthy controls along with 1.3% of the trials for the depressed group were removed because drift correction duration exceeded 7000ms. Additionally, 2.4% of trials for the healthy controls and 2.2% of trials for the depressed participants were discarded due to the fixation times to the target exceeding M ± 3SDs. Overall 11.0% of the trials for the healthy controls along with 11.5% of the trials for the depressed participants were excluded.

As in Study 1, we quantified the reward interference by calculating the extent to which fixation time to the target was prolonged (FTT _large_ - FTT _small_), the extent to which percentage of trials involving gaze directed at distractors was increased (PGD _large_ - PGD _small_), the extent to which percentage of trials where the first saccade went toward the distractor/target location was increased/decreased (SPD _large_ -SPD _small_/SPT _small_ - SPT _large_), and the extent to which the saccade latency that the first saccade endpoint was the location of the distractor/target was decreased/increased (SFD_small_ - SFD_large/_SFT_large_ - SFT_small_) when large-value distractors occurred compared with small-value distractors. Independent-samples t-tests toward these eye-tracking indicators were used to examine the difference in attentional resources to be captured by social-value distractors between the two groups. Repeated-measures ANOVAs with the group (depressed, control) as the between-subjects factor and the distractor kind (large value, small value, absent) as the within-subjects factor were added to conduct the further analysis. In addition, result of HLM also attempted to reveal a linear effect of depression on attentional resources to be captured by social-value distractors. Besides, we delved further into the time course of oculomotor capture by social-value distractors, to disclose the mechanism of the oculomotor capture.

### 3.2 Results

#### 3.2.1 Fixation time to target

Regarding the extent to which fixation time to the target was prolonged when the large-value distractors occurred compared with the small-value distractors (i.e., FTT _large_ - FTT _small_), the independent-samples T test showed no significant difference between the depressed group and the control group, *t* < 1. The results of 2(group:depressed, control)×3(distractor kind: large value, small value, absent) repeated-measures ANOVA revealed that the distractor kind was the only factor which showed a significant main effect, *F*(1.48, 158.71)=35.20, *p <*.001, η*_p_,^2^* = .248. LSD test indicated that the fixation time to the target were significantly delayed under the large-value distractor condition, separately compared with the conditions of small-value distractor and the distractor absent [large value: *M* = 401ms, *SEM =* 7.5ms, 95%CI:(386ms, 416ms); small value: *M* = 392ms, *SEM =* 7.3ms, 95%CI:(378ms, 407ms); absent: *M* = 365ms, *SEM =* 8.4ms, 95%CI:(349ms, 382ms)], large value *vs*. small value: *p=*.003, large value *vs*. absent: *p <*.001. Furthermore, when small-value distractors were present, the fixation time to target occurred later compared with that when the distractors were absent, *p <*.001. Neither the main effect of group nor the interaction between the two factors was significant, *Fs* < 1.

#### 3.2.2 Percentage of trials involving gaze directed at distractors

We then focused on the percentage of trials where participants directed their gaze toward the large- and small-value distractors to assess the extent to which oculomotor behavior could be influenced by distractors related to social reward. Regarding the extent to which percentage of trials involving gaze directed at distractors was increased when the large-value distractors occurred compared with small-value distractors (i.e., PGD _large_ - PGD _small_), the independent-samples T test revealed that the depressed group were less likely to be attracted by the large-value distractors compared with the control group, *t*(107) = 2.44, *p =*.016, Cohen’s *d =*.468. From the results of ANOVA with the factors of distractor kind(large value, small value, absent) and group(depressed, control), the distractor kind had a significant main effect, *F*(1, 107)=7.04, *p=*.009, η*_p_,^2^* = .062. The percentage of trials involving gaze directed at large-value distractors was greater than that at small-value distractors. Group’s main effect was not statistically significant, *F<*1. The interaction of group and distractor kind was also significant, *F*(1, 107)=5.96, *p =*.016, η*_p_,^2^* = .053. For the control group, the percentage of trials involving gaze directed at large-value distractors were more than that at small-value distractors, *p <*.001, while for the depressed group, frequency for gazes at large-value distractors did not obviously differ from that at small-value distractors, *p* = .882.

**Figure 4:**
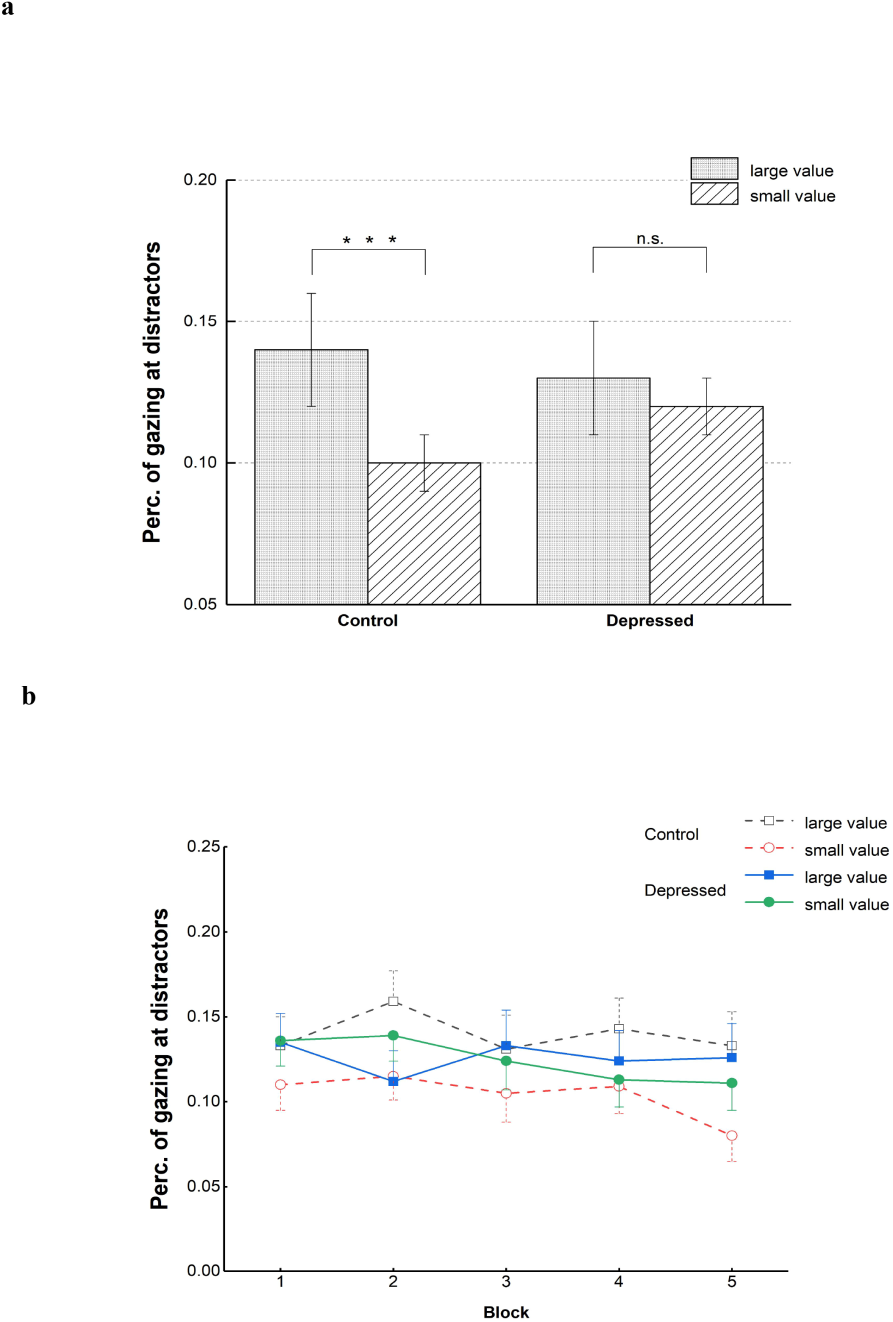
Percentages of trials involving gaze directed at distractors of two groups under two distractor-kind conditions **(a)** and mean percentages across five blocks **(b)**. ***p<.001, n.s., insignificant. Error bars denote standard errors.

We also performed an exploratory analysis to compare the extent that distractors related to social reward could influence the oculomotor behavior between the two groups across various blocks. An ANOVA was used to examine the percentage of trials involving gaze directed at distractors, considering the factors of group(depressed, control), distractor kind(large value, small value), and block(1-5). Similar to the above results, obvious interaction of distractor kind and group was found, *F*(1, 107)=5.34, *p =*.023, η*_p_,^2^* = .048. The distractor kind also had a significant main effect, *F*(1, 107)=6.37, *p=*.013, η*_p_,^2^* = .056. The main effects of block and group were both not statistically significant, block: *F*(3.28, 350.80)=1.40, *p =*.239, group: *F <* 1. No significant interaction of block and group was found, as the interaction of block and distractor kind, block*group: *F <* 1, block*distractor kind: *F*(4, 428)=1.36, *p =*.246. The three-way interaction between distractor kind, block, and group was also not significant, *F*(4, 428)=1.46, *p =*.213. The factor of block did not affect oculomotor capture by distractors related to social reward in both the control and depressed groups. **Figure 4** illustrates the average percentages of trial where participants gazed at the distractor under both large- and small-value conditions for the two groups.

#### 3.2.3 First saccade ending point

We also focused on the destination of the first saccade to uncover the difference in early influence of distractors related to social reward on oculomotor behavior between the two groups. The location of the target or distractor was defined as the saccade destination when the saccade endpoint deviated from the target or distractor center less than 30° (51). Concerning the percentage for trials where the first saccade went toward the distractor location(abbr. perc. of first saccade to the distractor), regarding the extent to which percentage of trials where the first saccade went toward the distractor was increased when the large-value distractors occurred compared with small-value distractors (i.e., SPD _large_ -SPD _small_), the independent-samples t-test indicated a weaker reward-interference effect for the depressed group than that for the control group, *t*(107) = 2.54, *p = .*013, Cohen’s *d =*.486. From the results of ANOVA with the factors of distractor kind(large value, small value, absent) and group(depressed, control), the main effect of distractor kind was significant, *F*(1, 107)=6.80, *p =*.010, η*_p_,^2^* = .060. A greater number of first saccades were directed toward large-value distractors than small-value distractors. The group did not had statistically significant main effect, *F<*1. There was a significant interaction between group and distractor kind, *F*(1, 107)=6.45, *p =*.013, η*_p_,^2^* = .057. For healthy controls, higher perc. of first saccade to the distractor under the large-value condition than the small-value condition was found, *p <*.001. While for the depressed group, no significant difference was found between the two distractor-kind conditions, *p* = .962.

**Figure 5:**
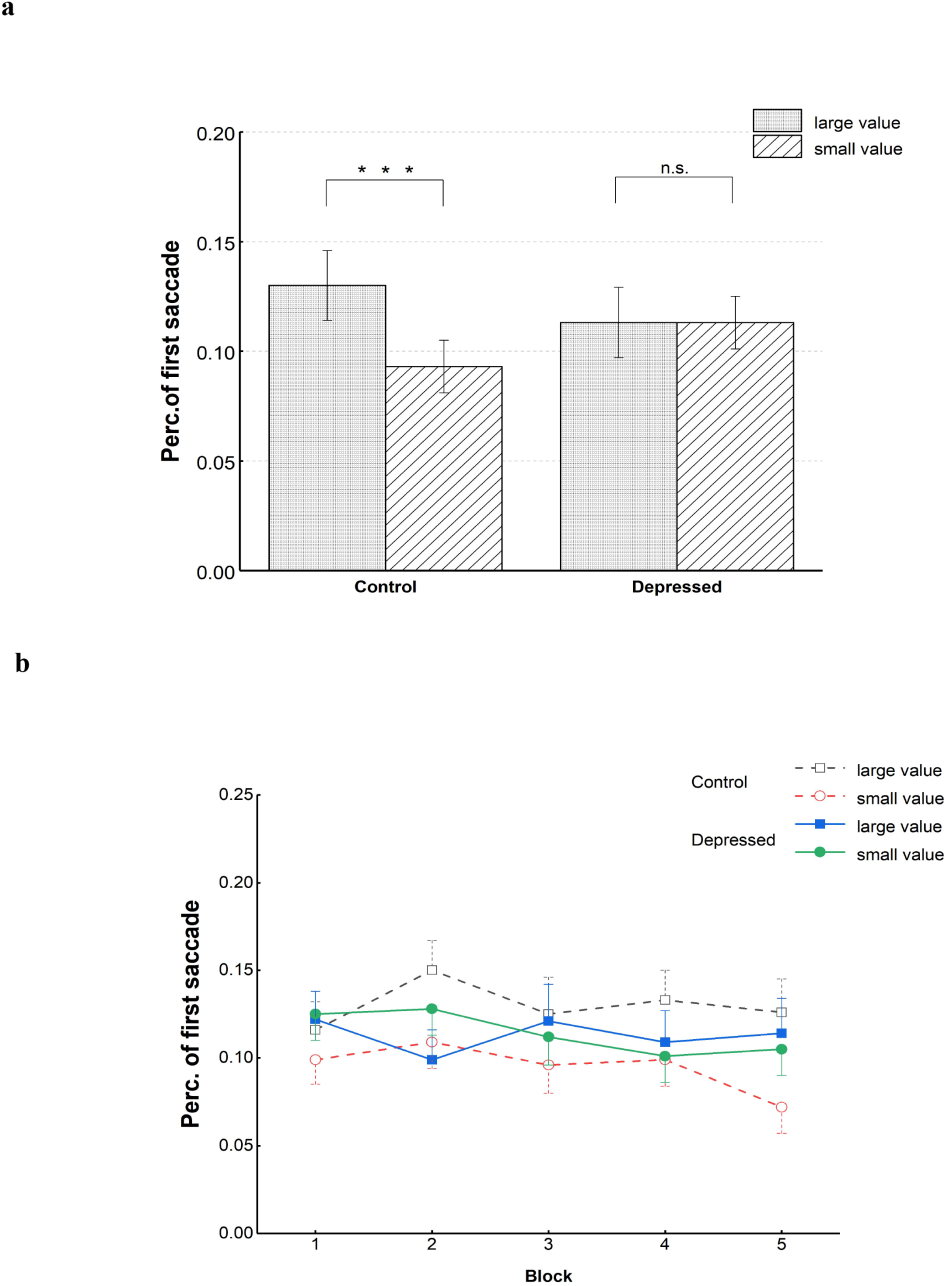
Percentages of trials where the first saccade ending point was the location of the distractor under various distractor-kind conditions for the two groups **(a)** and mean percentages across five blocks **(b)**. ***p<.001, n.s., insignificant. Error bars all denote standard errors.

Regarding the analysis under different blocks, the results for the ANOVA with the factors of group(depressed, control), distractor kind(large value, small value) and block(1-5) also showed that the distractor-kind’s main effect as well as the interaction of distractor kind and group were significant, distractor kind: *F*(1, 107)=5.57, *p =*.020, η*_p_,^2^* = .049, distractor kind*group: *F*(1, 107)=6.36, *p =*.013, η*_p_,^2^* = .056. Other main effects and interactions were all not significant, block: *F*(3.32, 355.38)=1.32, *p =*.266, group: *F* < 1, block*group: *F*(3.32, 355.38)=1.58, *p =*.189, distractor kind*block: *F*(4, 428)=1.32, *p =*.261, distractor kind*block*group: *F*(4, 428)=1.34, *p =*.253. **Figure 5** illustrates the average perc. of first saccade to the distractor for each group across various distractor-kind conditions.

Concerning the percentage for trials where the first saccade directed at the target (abbr. perc. of first saccade to the target), regarding the extent to which percentage of trials where the first saccade went toward the target was decreased when the large-value distractors occurred compared with small-value distractors (i.e., SPT_small_ -SPT_large_), the independent samples t-test demonstrated that there was no significant difference between the two groups, *t*(107) = 1.16, *p* = .247. From the results of ANOVA with the factors of distractor kind(large value, small value, absent) and group(depressed, control), the significant effect of distractor kind was found, *F*(1.60, 171.50)=67.20, *p <*.001, η*_p_,^2^* = .386. The LSD test indicated an obvious influence of distractors related to social reward on the perc. of first saccade to the target. The case for first saccade going toward the target under the condition of large-value distractors was less frequent than those under the conditions of small-value distractors and distractor absent respectively, large value *vs*. small value: *p = .*005, large value *vs*. absent: *p <*.001. Compared to the condition of distractor absent, the frequency of first saccade going toward the target also reduced as a small-value distractor was present, *p <*.001. The group effect was not statistically significant, *F* < 1.

Significant interaction between the two factors was found, *F*(1.60, 171.50)=3.97, *p =*.029, η*_p_,^2^* = .036. In healthy controls, the frequency for first saccade going toward the target in the large-value condition was less than those in the small-value and distractor-absent conditions respectively, large value *vs*. small value: *p = .*016, large value *vs*. absent: *p <*.001. Besides, lower perc. of first saccade to the target was found in the small-value condition than the distractor-absent condition, *p <*.001. However, for depressed participants, no significant difference in the perc. of first saccade to the target was observed between the large- and small-value conditions, *p* = .560. The first saccade going toward the target was more frequent under the distractor-absent condition compared with both large- and small-value conditions, *ps <*.001. On the difference for the influence of large-value distractors in the two groups, we could see that the results was not consistent between the analyses of independent-samples t-test and ANOVA. Therefore, we could not simply draw a conclusion about abnormal effect of social reward on the frequency of target as the first saccade destination among individuals with depressive symptoms. Regarding the results of ANOVA, it was possible that the random noise was greater in the depressed group, which led to the presentation of no obvious reward effect. See **Table 4** for the mean perc. of first saccade to the distractor/target from various distractor-kind conditions of the two groups.

**Table 4:** Mean percentages of trials involving gaze directed at distractors(%) and mean percentages of trials where the location of distractor/target was as the first saccade destination(%) under different distractor-kind conditions for the depressed and control groups (standard errors in parentheses, 95% confidence interval following the comma).

Indicators	Group	Large-value	Small-value	Absent
Perc. of gazing at distractors	Control	0.14(0.02), 0.11-0.17	0.10(0.01), 0.08-0.13	N/A
	Depressed	0.13(0.02), 0.09-0.16	0.12(0.01), 0.10-0.15	N/A
Perc.of first saccade to the distractor	Control	0.13(0.02), 0.10-0.16	0.09(0.01), 0.07-0.12	N/A
	Depressed	0.11(0.02), 0.08-0.15	0.11(0.01), 0.09-0.14	N/A
Perc. of first saccade to the target	Control	0.40(0.02), 0.35-0.44	0.43(0.02), 0.39-0.47	0.56(0.02), 0.52-0.60
	Depressed	0.42(0.02), 0.38-0.46	0.43(0.02), 0.39-0.47	0.52(0.02), 0.47-0.56

In order to demonstrate the effect of depression on the gaze to be captured by social-value distractors continuously, two HLMs employing restricted maximum likelihood estimation were analyzed, one with the percentage of trials involving gaze directed at distractors serving as the dependent variable and the other with the perc. of first saccade to the distractor as the dependent variable. The focus was placed on the fixed effect for the interaction of BDI-II score and distractor kind(large value, small value). The score on the anxiety subscale of the SCL-90-R scale, which reflected the level of anxiety, was selected as the covariate. In two HLMs, multicollinearity were both not found between the variables (VIFs ≤ 1.62).

As in Study 1, for each participant, in each block, trials for two distractor-kind conditions were collapsed into two separate observations. Thus, at five blocks, ten observations was provided from each participant, with half of which belonging to the condition of large-value and other half belonging to the condition of low-value. The participants were regarded as random factors, contributing to the accounting for the variance within the subjects. Overall, the total number of participants in this study was 109, and the total number of observations was 1,090. These met the sample size for investigating the interaction between cross-level variables, as outlined in the work of Hox et al. (52). In the HLM with the percentage of trials involving gaze directed at distractors as the dependent variable, as our expected, the results showed that a substantial positive moderating effect for the score of BDI-II on the predictive relationship between distractor kind and percentage of trials involving gaze directed at distractors was identified, *standardized β* = 0.07(95%CI: 0.00, 0.13), *t*(105.53)=2.00, *p=*.048. **Table 3** presents the effect of variables in the two HLMs.

#### 3.2.4 First saccade latency

We used a repeated-measures ANOVA with the factors of group(control, depressed) and distractor kind(large value, small value, absent) to analyze the latency for first saccade of the two groups across different distractor-kind conditions. No significant main effects of group and distractor kind were found, group: *F*(1, 107)=1.76, *p =*.187, distractor kind: *F<*1. Two factor’s interaction was also not significant, distractor kind * group: *F*(1.58, 169.35)=2.14, *p =*.132.

Regarding the extent to which the saccade latency that the first saccade endpoint was the location of the distractor was decreased (SFD_small_ - SFD_large_) when large-value distractors occurred compared with small-value distractors, the independent samples t-test demonstrated that no significant difference between the two groups was found, *t <* 1. From the results of ANOVA with the factors of distractor kind(large value, small value, absent) and group(depressed, control), only the distractor kind showed a significant main effect, *F*(1, 105)=4.82, *p =*.030, η*_p_,^2^* = .044. When the first saccade ending point was the large-value distractor, the latency of the first saccade was significantly shorter than that when the ending point of the first saccade was the small-value distractor. Group’s main effect and the interaction between the two factors were both not significant, group: *F*(1, 105)=3.01, *p =*.086, distractor kind*group: *F <* 1.

Regarding the extent to which the saccade latency that the first saccade endpoint was the location of the target was increased(SFT_large_ - SFT_small_) when large-value distractors occurred compared with small-value distractors, the independent samples t-test also demonstrated that no significant difference between the two groups was found, *t <* 1. From the results of ANOVA with the factors of distractor kind(large value, small value, absent) and group(depressed, control), significant main effect of distractor kind was found, *F*(1.71, 182.60)=14.18, *p <*.001, η*_p_,^2^* = .117. The LSD test indicated shorter saccade latency was found in the absence of a distractor compared with when either a large- or small-value distractor was present, *ps <*.001. Saccade latency to the target showed no significant difference between the conditions of large-and small-value, *p=*.580. Group’s effect and the interaction were both not significant, group: *F*(1, 107)=1.99, *p =*.162, distractor kind*group: *F*(1.71, 182.60)=1.51, *p =*.223.

#### 3.2.5 Time course of oculomotor capture by distractors

We focused on analyzing the data for the control group who was clearly captured by the social-value distractors. Each healthy control’s first saccade latency was categorized into 4 equal intervals based on quartiles separately for large- and small-value conditions. The percentage of first saccades directed toward the distractor’s location at each interval was calculated to disclose the time course of oculomotor capture by distractors related to social reward.

**Figure 6:**
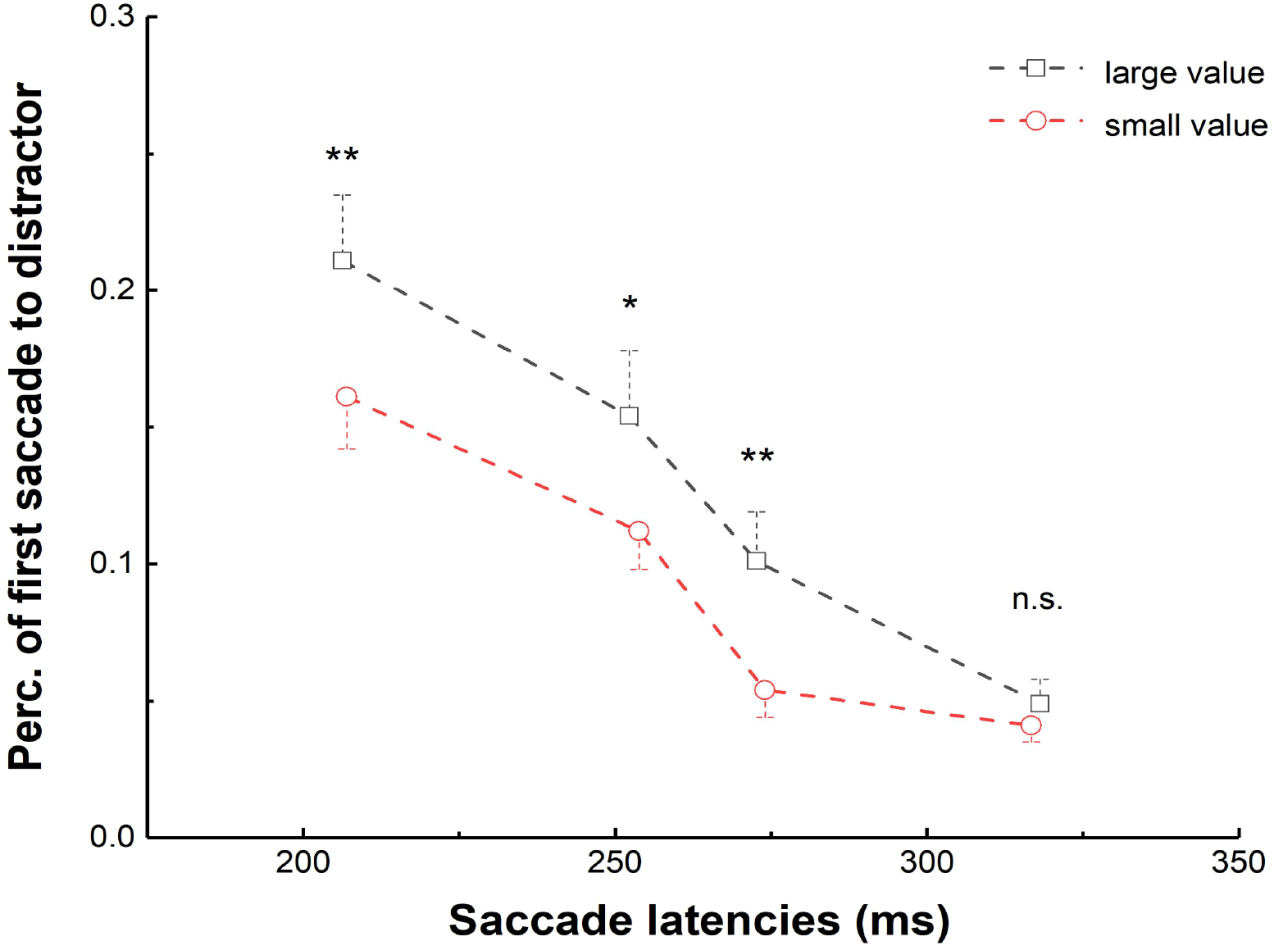
Mean percentages of trials where the first saccade ending point was the distractor across four equal intervals of first saccade latency under different distractor-kind conditions. Error bars denote standard errors.*p < 0.05, **p < 0.01, n.s., insignificant.

A two-factor ANOVA was conducted involving factors of distractor kind(large value, small value) and first saccade-latency interval(1–4) to analyze the perc. of first saccade to the distractor under different distractor-kind conditions across the four first saccade-latency intervals. Same as the distractor kind, the interval also had a significant main effect, distractor kind: *F*(1, 54)=11.86, *p =*.001, η*_p_,^2^* = .180, interval: *F*(1.95, 105.22)=35.67, *p <*.001, η*_p_,^2^* = .398. With longer saccade latency, the probability of the distractor location as the first saccade destination decreased. In addition, significant interaction between the distractor kind and interval was found, *F*(3, 162)=3.48, *p =*.017, η*_p_,^2^* = .061. At intervals 1–3, the frequency for the large-value distractors as the first saccade destination was notably greater than that for the small-value distractors, interval 1,3: *ps=*.001, interval 2: *p=*.020. While at interval 4, no significant difference was found for the frequency of the distractor as the first saccade destination across different conditions of distractor kind, *p=*.276. **Figure 6** illustrates the time course of oculomotor capture by distractors.

### 3.3 Discussion

The results of the first saccade latency showed that compared with the presentation of small-value distractors or the absence of reward-related distractors, when the large-value distractors were presented, no significant delay was found in the initial saccade. Stimuli with strong emotional intensity will force individuals to undergo a process of behavioral slowdown, in order to adapt to emotional cognitive situations (53, 54). Our results indicated that a reduction of the first saccadic flexibility caused by the emotional information was not obvious in current study. For the results of fixation time to the target and other related temporal indicators, it was reasonable to believe that the delayed fixation to the target in the presence of large-value distractors more tended to reflect the phenomenon of attentional capture by the distractors rather than the behavioral adjustment.

It was worth noting that, from the indicator of fixation time to the target, we actually did not observe an obvious moderate effect of depression level on the attentional capture by social-value distractors, which was contrast to the findings for the measurement of keypress-response time in Study 1. This could be related to psychomotor disturbance for individuals with depressive symptoms, and negative emotional effect was more pronounced in responses that involved more mental processings and physical coordination. As outlined in the Diagnostic and Statistical Manual of Mental Disorders, Fifth Edition (DSM-5), individuals with depressive symptoms typically exhibit daily psychomotor retardation symptoms stemming from mood disorders, characterized by slowed or reduced movement (1). Given the rising prevalence of psychomotor impairment among individuals with depressive symptoms, researchers and therapists have been developing and refining quantitative clinical assessment tools (55, 56).

The difference in response patterns between the eye movement task and the keypress-response task was precisely the key inducement that psychomotor disorders had a distinct influence on the findings of Studies1 and 2. In the eye movement task, the participants only needed to fix their gaze on the target, which was simple and easy to perform. While in the manual keypress-response task of Study 1, the response involved hand-eye coordination and other mental processings. Due to the influence of negative emotional disorders on these, the response process would be slowed, which finally covered up the effect of reward interference. This further led to the finding in Study1 that individuals with depressive symptoms showed less attentional bias toward social-value distractors than healthy controls. It could be said that the psychomotor disturbance made the characteristic of individuals with depressive symptoms, which they showed insufficient attention bias toward social-value stimuli, more prominent in the keypress-response task. Researchers had previously emphasized the influence of motor characteristics on keypress response (57). Which aligned with this, the findings of our study clearly reflected the role of manual response feature resulting from emotional disorders on the keypress-response. Our results of keypress-response task was also consistent with those in the studies of Anderson et al. (13, 14) and Brailean et al. (15). They conducted the tasks related to keypress-response as well and focused on the general forms of the reward in the experimental studies, points/money. They all found that compared with the healthy controls, the influence of reward-related information on the task performance for the individuals with depressive symptoms was very limited. Whether it is the reward based on the social feedback or not, through the data collection method of keypress-response, it was found that the interference effect of reward information in the individuals with depressive symptoms was significantly lower than that in the healthy controls.

Alternatively, the distribution of individual depression levels in the depressed group may explain the lack of significant differences in some eye movement indicators between the depressed group and the control group. In Study 2, unlike the high proportion of moderately to severely depressed participants in Study 1, nearly half of the depressed participants were in the mild depression category(i.e., 24 members, 44% of the total number of the depressed group). It is likely that in terms of responding to social reward information, individuals with mildly depressive symptoms remain relatively normal eye movement characteristics and no significant differences exist in the measurement of related eye-tracking indicators between individuals with mildly depressive symptoms and the healthy controls. As a result, no significant differences between the entire depressed group and the control group for these indicators(i.e., fixation time to target, first saccade latency to the value-related distractor/target when it was the first saccade ending point, etc) were detected. This is in line with the findings of the study from Monéger et al. (58), which revealed that certain attentional bias patterns or degrees varied among individuals with different levels of depressive symptoms. In other words, Study 2, for including a significant number of individuals with mild depressive symptoms, has found some intact eye movement response characteristics toward social reward information in those individuals with milder depressive symptoms. Future research could increase the participation rate of individuals with moderate to severe depressive symptoms to conduct a more in-depth and structured exploration of the differences in eye movement characteristics in response to social reward information between individuals with depressive symptoms and healthy controls.

The differences between the depressed group and the control group were mainly manifested in the results of the spatial indicators, which revealed the deficit in overt attentional capture by social-value distractors among individuals with depressive symptoms. Regarding overt attention, distractors related to social reward exhibited fewer advantage in oculomotor capture among individuals with depressive symptoms than healthy controls. Furthermore, for the percentage of trials involving gaze directed at distractors, by establishing a hierarchical linear model, we also revealed that high BDI-II scores led to an obvious alteration in the bias of oculomotor capture by social-value distractors. This demonstrated the significant effect of depressive status on overt attentional capture by stimuli related to social reward from the perspective of continuous analysis.

Regarding the time course of oculomotor capture by distractors, our results showed that, bias of oculomotor capture by social-value distractors mainly resulted from the individual’s insufficient proactive visual inhibition of the interference rather than the poor performance of reactive suppression. In other words, social-value distractors had potential priority in attentional capture. From the results, compared with the low-value distractors, the locations of large-value distractors were more tended to be selected as the first saccade destinations at intervals 1–3. No significant difference was observed in the tendency to be the first saccade ending point between the large- and small-value distractors at interval 4. It could be seen that the advantage of attracting saccades for large-value distractors mostly occurred at the occasion of short saccade latency. This finding aligned with those of prior researches. Reward information often captures gazes during early visual selection, and this can only be mitigated through increased saccadic inhibition which manifested as the prolonged saccadic latency (59, 60, 80). In conclusion, by exploring the time course of oculomotor capture, from the perspective of visual inhibition, we initially inferred the explanation of how social reward information captured overt attention. However, future research should conduct more rigorous and comprehensive analyses to refine the exploration of the related mechanisms.

## 4 General discussion

Our study, grounded in the attentional model for associative learning, examined the case that attentional resources were captured by distractors related to social reward among individuals with depression to contribute to the understanding for the atypical social-reward learning. Compared with the healthy controls, individuals with depressive symptoms were less likely to be disturbed by the distractors related to social reward in the manual keypress-response task. In addition, relying on the recording of eye movements, we revealed fewer advantage of social-value distractors in oculomotor capture among individuals with depressive symptoms than healthy controls. Such group of people exhibited a decrease in attentional resources to be captured by distractors related to social reward, which manifested as a type of social reward-learning disabilities. Researches on social-reward learning in individuals with depressive symptoms only indicated that their learning rate of behavioral responses linked to social-reward acquisition was significantly lower compared with those with typical development (8, 9). Our findings provided evidences for deficit in attentional resources to be captured by social-value stimuli among individuals with depressive symptoms. This enhanced our comprehension of social reward-learning disabilities by examining the attentional processing toward reward-conditioned stimuli.

From the perspective of associative-learning mechanisms, in our study, the social-reward related stimuli were task-irrelevant and only as distractors indicating that chances of obtaining the social reward by making correct and quick responses existed. As a result, we concentrated on the reward-conditioned stimuli with obvious signal value and identified a decrease in attentional resources to be captured by stimuli related to social reward in individuals with depressive symptoms under the framework of classical conditioning. It had been demonstrated that during the process of classical conditioning, individuals with depressive symptoms showed a deficit in attentional capture by reward-related conditioned stimuli with signal value (61). Our findings aligned with this and revealed the fact that individuals with depressive symptoms also showed an attentional impairment toward the conditioned stimuli related to social reward similarly within the framework of classical conditioning.

One prominent feature for social anhedonia among individuals with depressive symptoms is low responsiveness to social rewards compared with healthy controls, a trait revealed by a series of neurophysiological studies. From ERPs studies, researchers revealed that, compared with healthy controls, individuals with depressive symptoms had attenuated RewP to social acceptance (21). This indicated that the initial automatic differentiation of reward versus neutral outcomes was less obvious among individuals with depressive symptoms. Following the presentation of the social incentive gesture, individuals with depressive symptoms exhibited a lower amplitude for feedback-P3(fb-P3) compared with healthy controls (25, 62). This reflected that, for individuals with depression, social reward had lower outcome salience, and correspondingly they showed lower intensity to make a response to the feedback of social rewards. A review of fMRI studies on social-reward processing also revealed that individuals with depression showed reduced activation in the prefrontal reward regions, specifically the medial prefrontal cortex and orbitofrontal cortex, in response to social rewards (63). From the perspective of neuroimaging, it was proved that increased depression significantly reduced the affective processing of social rewards. All these studies support low responsiveness to social rewards in depressed individuals. Our findings are consistent with the characteristic of lower social-reward responsiveness in individuals with depression. There is reason to believe that lower responsiveness to social rewards makes the related conditioned stimuli insufficient for inducing attentional bias, thus leading to a decrease in attentional resources to be captured by stimuli related to social reward among those with depressive symptoms.

In individuals with depressive symptoms, deficit in attentional resources to be captured by social-value stimuli were consistent with atypical neural activity when reward cues were perceived. Researches indicated that depressed individuals showed decreased neural activity at the cortex of striatum and anterior cingulate, along with diminished frontostriatal connectivity, in response to reward-related conditioned stimuli compared with healthy controls (64–66). After the occurrence of prediction error, individuals with depression showed reduced neutral activity in the striatum and non-brainstem areas compared with healthy controls when the conditioned stimulus was re-presented (67, 68). All of these demonstrate that individuals with depressive symptoms exhibit poor neural activity in related cortices when processing reward-related conditioned stimuli.

Regarding social-reward processing, from an ERP study, researchers found that, individuals with depressive symptoms showed a low magnitude for the component of CNV and a low hit rate to large-magnitude social-reward cues than healthy controls (18). Additionally, individuals with melancholic major depressive disorder also exhibited a reduced magnitude of the N2 component in response to the social- reward cues (69). Poor behavioral preparation induced by social-reward cues has been observed in individuals with depression. About this, our findings provide an explanation that individuals with depressive symptoms have less attentional resources to be captured by stimuli related to social reward. Disorders of attentional processing to the social-reward related stimuli could lead to a small effect of the stimuli on behavioral preparation.

Based on the competitive integration model, the saccade map theory is a good explanation for overt visual search pattern. The theory posited that during a process of visual search, a saccade is triggered to a location when the saccade activation value of the location surpasses a certain threshold (35, 70). Our findings had clarified the effects of social-reward information on visual search. Stimuli locations with greater social-reward value exhibited increased value of saccade-activity. This makes the locations attractive and more frequent as saccade and even first saccade destinations. Furthermore, the status of individuals’ depressive symptoms significantly moderated the relationship between the social value of the stimuli and the saccadic-activation values of their positions. The saccadic-activation value for the location of social-reward stimuli was lower for individuals with depressive symptoms than healthy controls. Consequently, among individuals with depressive symptoms, social-value stimuli locations have less advantage in attracting saccades.

Apart from the depression, the exploration of social reward processing also existed in individuals with autism and social anxiety disorders. Social impairment has long been recognized as the core disorder of Autism Spectrum Disorder(ASD) (1). Based on the social motivation hypothesis, researchers demonstrated at both behavioral and neurophysiological levels that the individuals with ASD had a lower sensitivity to social rewards and were less inclined to make efforts or made less efforts to obtain them (71–75). From the process of reward learning, the learning rate of social reward related stimuli for individuals with ASD was also lower than that for the healthy controls (76, 77). Besides, as with the obvious social impairments, the individuals with social anxiety disorder also exhibited significant deficit in the processing of social rewards. Studies at the neurophysiological level revealed that people with social anxiety exhibited significantly lower reactivity and sense of anticipation to social rewards (23, 78, 81). All of these suggested that the decline of attentional processing toward social reward related stimuli revealed in our study may not be unique to people with depressive symptoms, but rather a common phenotypic feature among abnormal populations with social cognitive impairments. Thus, it was meaningful to expand our research topic under the cross-diagnostic context and deeper explore the underlying mechanisms.

Our study innovatively explored reward-learning impairments in individuals with depressive symptoms from an attentional perspective, but several limitations remained. Firstly, majority of participants experiencing depression were not definitively diagnosed with major depressive disorder. Researchers believed that mild to severe depression developed on a continuum, and a quantitative difference rather than qualitative change existed in cognitive and emotional abnormalities between individuals with clinical and non-clinical depression (79). Nevertheless, the replication of our findings into clinical samples remains meaningful.

Secondly, because the participants were undergraduate students, our findings were clearly more applicable to young adults with depressive symptoms. Therefore, caution should be exercised when generalizing our conclusions to other age groups. Researchers can attempt to explore the developmental trend of attentional resources to be captured by social-value stimuli in individuals with depressive symptoms from pueritia to old age. In addition to this, our participants are all from China. The high homogeneity of the sample’s culture and demographic background may affect the generalizability of the conclusions, as individuals from different culture backgrounds could have different interpretations of the social stimuli. Future studies can be conducted in populations with other cultural backgrounds to verify these findings.

Thirdly, shortcomings presented in the continuous measurement of attentional processing toward the stimuli related to reward. Owing to the specific settings of the experimental procedure, our observation for attentional resources to be captured by stimuli related to social reward focused on the phase before the response to the target. The social-value stimuli disappeared after the response was completed. Studies found that individuals with depression exhibited atypical neural electrical activity in anticipation processing of social rewards after a behavioral response and the consummatory processing of social rewards (20, 21). In these phases, the phenomenon of attentional capture by stimuli related to social reward are also likely to be abnormal among individuals with depressive symptoms. Future studies should enhance the ongoing presentation of the stimuli and offer a more thorough examination in the attentional resources to be captured by social-value stimuli for individuals with depression.

Finally, our research still lacked in stimulus validation. Although the utilization of smiling and peaceful facial expressions as proxies for high- and low- social rewards was supported by the prior work, we merely assumed that the difference existed in the reward magnitude between smiling and peaceful facial expressions, while the differences in other facial feature dimensions such as arousal, valence, and familiarity between the expressions were not well considered. Moreover, the individual or cultural difference in interpreting facial expressions may also influence the results. Future researchers should implement stricter control in the validation of stimulus presentation and the manipulation of social rewards.

## 5 Conclusion

Under the classical conditioning, individuals with depressive symptoms displayed a deficit in attentional resources to be captured by social-value stimuli. Specifically, when social-value stimuli were as obvious distractors in the task, the interference of them was less toward individuals with depressive symptoms than healthy controls in the keypress-response task. In addition, social reward information showed less influence on oculomotor behavior for individuals with depressive symptoms. Our study, by observing the attentional bias toward conditioned stimuli, revealed one manifestation of the learning disorder involving social reward among individuals with depressive symptoms.

## Data Availability

The raw data supporting the conclusions of this article will be made available by the authors, without undue reservation.

## References

[1] American Psychiatric Association . Diagnostic and statistical manual of mental disorders. 5th. Washington, DC: The American Psychiatric Association (APA (2013).

[2] Enneking V Krüssel P Zaremba D Dohm K Grotegerd D Förster K . Social anhedonia in major depressive disorder: A symptom-specific neuroimaging approach. Neuropsychopharmacology. (2019) 44:883–9. doi: 10.1038/s41386-018-0283-6, PMID: 30607014 PMC6461766

[3] Gandhi A Mote J Fulford D . A transdiagnostic meta-analysis of physical and social anhedonia in major depressive disorder and schizophrenia spectrum disorders. Psychiatry Res. (2022) 309:114379. doi: 10.1016/j.psychres.2021.114379, PMID: 35123252

[4] Sagud M Tudor L Šimunić L Jezernik D Madžarac Z Jakšić N . Physical and social anhedonia are associated with suicidality in major depression, but not in schizophrenia. Suicide Life-Threatening Behav. (2021) 51:446–54. doi: 10.1111/sltb.12724, PMID: 33314250

[5] Hyldelund NB Byrne DV Chan RC Andersen BV . The Relationship between social anhedonia and perceived pleasure from food—an exploratory investigation on a consumer segment with depression and anxiety. Foods. (2022) 11:3659. doi: 10.3390/foods11223659, PMID: 36429251 PMC9689578

[6] Donnelly BM Hsu DT Gardus J Wang J Yang J Parsey RV . Orbitofrontal and striatal metabolism, volume, thickness and structural connectivity in relation to social anhedonia in depression: A multimodal study. NeuroImage: Clin. (2024) 41:103553. doi: 10.1016/j.nicl.2023.103553, PMID: 38134743 PMC10777107

[7] Berridge KC Robinson TE Aldridge JW . Dissecting components of reward: ‘liking’, ‘wanting’, and learning. Curr Opin Pharmacol. (2009) 9:65–73. doi: 10.1016/j.coph.2008.12.014, PMID: 19162544 PMC2756052

[8] Frey AL Frank MJ McCabe C . Social reinforcement learning as a predictor of real-life experiences in individuals with high and low depressive symptomatology. psychol Med. (2021) 51:408–15. doi: 10.1017/S0033291719003222, PMID: 31831095 PMC7958481

[9] Hammond D Xu P Ai H Van Dam NT . Anxiety and depression related abnormalities in socio-affective learning. J Affect Disord. (2023) 335:322–31. doi: 10.1016/j.jad.2023.05.021, PMID: 37201901

[10] Anderson BA . Relating value-driven attention to psychopathology. Curr Opin Psychol. (2021) 39:48–54. doi: 10.1016/j.copsyc.2020.07.010, PMID: 32818794 PMC7854796

[11] Le Pelley ME Mitchell CJ Beesley T George DN Wills AJ . Attention and associative learning in humans: An integrative review. psychol Bull. (2016) 142:1111–40. doi: 10.1037/bul0000064, PMID: 27504933

[12] Mackintosh NJ . A theory of attention: Variations in the associability of stimuli with reinforcement. psychol Rev. (1975) 82:276–98. doi: 10.1037/h0076778

[13] Anderson BA Leal SL Hall MG Yassa MA Yantis S . The attribution of value-based attentional priority in individuals with depressive symptoms. Cognitive Affective Behav Neurosci. (2014) 14:1221–7. doi: 10.3758/s13415-014-0301-z, PMID: 24874421 PMC4221358

[14] Anderson BA Chiu M DiBartolo MM Leal SL . On the distinction between value-driven attention and selection history: Evidence from individuals with depressive symptoms. Psychonomic Bull Rev. (2017) 24:1636–42. doi: 10.3758/s13423-017-1240-9, PMID: 28210998 PMC5559347

[15] Brailean AM Koster EH Hoorelbeke K De Raedt R . Attentional modulation by reward and punishment cues in relation to depressive symptoms. J Behav Ther Exp Psychiatry. (2014) 45:351–9. doi: 10.1016/j.jbtep.2014.03.003, PMID: 24727341

[16] Hertz-Palmor N Rozenblit D Lavi S Zeltser J Kviatek Y Lazarov A . Aberrant reward learning, but not negative reinforcement learning, is related to depressive symptoms: An attentional perspective. psychol Med. (2024) 54:794–807. doi: 10.1017/S0033291723002519, PMID: 37642177

[17] Fussner LM Mancini KJ Luebbe AM . Depression and approach motivation: Differential relations to monetary, social, and food reward. J Psychopathol Behav Assess. (2018) 40:117–29. doi: 10.1007/s10862-017-9620-z

[18] Zhang D Shen J Bi R Zhang Y Zhou F Feng C . Differentiating the abnormalities of social and monetary reward processing associated with depressive symptoms. psychol Med. (2022) 52:2080–94. doi: 10.1017/S0033291720003967, PMID: 33143780

[19] Taylor CT Stein MB Simmons AN He F Oveis C Shakya HB . Amplification of positivity treatment for anxiety and depression: A randomized experimental therapeutics trial targeting social reward sensitivity to enhance social connectedness. Biol Psychiatry. (2024) 95:434–43. doi: 10.1016/j.biopsych.2023.07.024, PMID: 37607657 PMC12063735

[20] He Z Ao X Muhlert N Elliott R Zhang D . Neural substrates of expectancy violation associated with social feedback in individuals with subthreshold depression. psychol Med. (2022) 52:2043–51. doi: 10.1017/S0033291720003864, PMID: 33109293

[21] Hill KE Dickey L Pegg S Dao A Arfer KB Kujawa A . Associations between parental conflict and social and monetary reward responsiveness in adolescents with clinical depression. Res Child Adolesc Psychopathol. (2023) 51:119–31. doi: 10.1007/s10802-022-00949-7, PMID: 35852700 PMC9771890

[22] Kujawa A . Reduced reward responsiveness and depression vulnerability: Consideration of social contexts and implications for intervention. Psychophysiology. (2024) 61:e14528. doi: 10.1111/psyp.14528, PMID: 38263892 PMC11096075

[23] Nelson BD Jarcho JM . Neural response to monetary and social feedback demonstrates differential associations with depression and social anxiety. Soc Cogn Affect Neurosci. (2021) 16:1048–56. doi: 10.1093/scan/nsab055, PMID: 33942882 PMC8483280

[24] Freeman C Panier L Schaffer J Weinberg A . Neural response to social but not monetary reward predicts increases in depressive symptoms during the COVID-19 pandemic. Psychophysiology. (2023) 60:e14206. doi: 10.1111/psyp.14206, PMID: 36349469 PMC9878199

[25] Ait Oumeziane B Jones O Foti D . Neural sensitivity to social and monetary reward in depression: Clarifying general and domain-specific deficits. Front Behav Neurosci. (2019) 13:199. doi: 10.3389/fnbeh.2019.00199, PMID: 31649515 PMC6794449

[26] Palacios-Barrios EE Patel K Hanson JL . Early life interpersonal stress and depression: Social reward processing as a potential mediator. Prog Neuropsychopharmacol Biol Psychiatry. (2024) 129:110887. doi: 10.1016/j.pnpbp.2023.110887, PMID: 39492470

[27] Pegg S Ethridge P Shields GS Slavich GM Weinberg A Kujawa A . Blunted social reward responsiveness moderates the effect of lifetime social stress exposure on depressive symptoms. Front Behav Neurosci. (2019) 13:178. doi: 10.3389/fnbeh.2019.00178, PMID: 31447659 PMC6692494

[28] Olino TM Silk JS Osterritter C Forbes EE . Social reward in youth at risk for depression: A preliminary investigation of subjective and neural differences. J Child Adolesc Psychopharmacol. (2015) 25:711–21. doi: 10.1089/cap.2014.0165, PMID: 26469133 PMC4653819

[29] Le Pelley ME Pearson D Griffiths O Beesley T . When goals conflict with values: Counterproductive attentional and oculomotor capture by reward-related stimuli. J Exp Psychology: Gen. (2015) 144:158–71. doi: 10.1037/xge0000037, PMID: 25420117

[30] Anderson BA . The attention habit: How reward learning shapes attentional selection. Ann New York Acad Sci. (2016) 1369:24–39. doi: 10.1111/nyas.12957, PMID: 26595376

[31] Adam K Patel T Rangan N Serences JT . Classic visual search effects in an additional singleton task: An open dataset. J Cogn. (2021) 4:34. doi: 10.5334/joc.182, PMID: 34396037 PMC8323537

[32] Pearson D Le Pelley ME . Reward encourages reactive, goal-directed suppression of attention. J Exp Psychology: Hum Percept Perform. (2021) 47:1348–64. doi: 10.1037/xhp0000946, PMID: 34766819

[33] Geng JJ . Attentional mechanisms of distractor suppression. Curr Dir psychol Sci. (2014) 23:147–53. doi: 10.1177/0963721414525780

[34] Wolf C Lappe M . Top-down control of saccades requires inhibition of suddenly appearing stimuli. Attention Perception Psychophysics. (2020) 82:3863–77. doi: 10.3758/s13414-020-02101-3, PMID: 32803547 PMC7593282

[35] Godijn R Theeuwes J . Programming of endogenous and exogenous saccades: Evidence for a competitive integration model. J Exp Psychology: Hum Percept Perform. (2002) 28:1039–54. doi: 10.1037/0096-1523.28.5.1039, PMID: 12421054

[36] Gaspelin N Luck SJ . Inhibition as a potential resolution to the attentional capture debate. Curr Opin Psychol. (2019) 29:12–8. doi: 10.1016/j.copsyc.2018.10.013, PMID: 30415087 PMC6488460

[37] Wang B Theeuwes J . How to inhibit a distractor location? Statistical learning versus active, top-down suppression. Attention Perception Psychophysics. (2018) 80:860–70. doi: 10.3758/s13414-018-1493-z, PMID: 29476331

[38] Faul F Erdfelder E Buchner A Lang AG . Statistical power analyses using G*Power 3.1: tests for correlation and regression analyses. Behav Res Methods. (2009) 41:1149–60. doi: 10.3758/BRM.41.4.1149, PMID: 19897823

[39] Silvia PJ Allan WD Beauchamp DL Maschauer EL Workman JO . Biased recognition of happy facial expressions in social anxiety. J Soc Clin Psychol. (2006) 25:585–602. doi: 10.1521/jscp.2006.25.6.585

[40] Beck AT Steer RA Brown GK . Beck depression inventory. 2nd ed. San Antonio, TX: The Psychological Corporation (1996).

[41] Carney CE Ulmer C Edinger JD Krystal AD Knauss F . Assessing depression symptoms in those with insomnia: An examination of the beck depression inventory second edition (BDI-II). J Psychiatr Res. (2009) 43:576–82. doi: 10.1016/j.jpsychires.2008.09.002, PMID: 18954876 PMC2677199

[42] von Glischinski M von Brachel R Hirschfeld G . How depressed is “depressed”? A systematic review and diagnostic meta-analysis of optimal cut points for the Beck Depression Inventory revised (BDI-II). Qual Life Res. (2019) 28:1111–8. doi: 10.1007/s11136-018-2050-x, PMID: 30456716

[43] Derogatis LR Cleary PA . Confirmation of the dimensional structure of the SCL-90: A study in construct validation. J Clin Psychol. (1977) 33:981–9. doi: 10.1002/1097-4679(197710)33:4<981::AIDJCLP2270330412>3.0.CO;2-0

[44] Yu Y Wan C Zhao X Huebner ES Tan J Xu C . Undergraduate students’ norms for the Chinese version of the symptom check-List-90-R (SCL-90-R). BMC Public Health. (2020) 20:1588. doi: 10.1186/s12889-020-09689-z, PMID: 33087089 PMC7579932

[45] First MB Gibbon M Spitzer RL . Structured clinical interview for DSM-IV-TR Axis I disorders, Research Version, Non-Patient. New York, NY: Biometrics Research Department (2002). SCID-I/NP). (ed. N. Y. S. P. Institute.

[46] Carver CS White TL . Behavioral inhibition, behavioral activation, and affective responses to impending reward and punishment: The BIS/BAS scales. J Pers Soc Psychol. (1994) 67:319–33. doi: 10.1037/0022-3514.67.2.319

[47] Martinez AM Benavente R . The AR face database. In: CVC technical report 24 Purdue University, Indiana, America: CVC(Computer Vision center, Purdue University). (1998).

[48] Anderson BA . Social reward shapes attentional biases. Cogn Neurosci. (2016) 7:30–6. doi: 10.1080/17588928.2015.1047823, PMID: 25941868 PMC4654995

[49] Watson P Pearson D Theeuwes J Most SB Le Pelley ME . Delayed disengagement of attention from distractors signalling reward. Cognition. (2020) 195:104125. doi: 10.1016/j.cognition.2019.104125, PMID: 31751815

[50] Kreft IG . Are multilevel techniques necessary? An overview, including simulation studies. Los Angeles: California State University (1996).

[51] Pearson D Watson P Cheng PX Le Pelley ME . Overt attentional capture by reward-related stimuli overcomes inhibitory suppression. J Exp psychology: Hum Percept Perform. (2020) 46:489–501. doi: 10.1037/xhp0000728, PMID: 32191108

[52] Hox J Moerbeek M van de Schoot R . Multilevel analysis: techniques and applications. 3rd ed. New York: Routledge (2017).

[53] Müller S Rothermund K Wentura D . Relevance drives attention: ttentional bias for gain- and loss-related stimuli is driven by delayed disengagement. Q J Exp Psychol. (2016) 69:752–63. doi: 10.1080/17470218.2015.1049624, PMID: 25980956

[54] Clarke PJF MacLeod C Guastella AJ . Assessing the role of spatial engagement and disengagement of attention in anxiety-linked attentional bias: A critique of current paradigms and suggestions for future research directions. Anxiety Stress Coping. (2013) 26:1–19. doi: 10.1080/10615806.2011.638054, PMID: 22136158

[55] Bingham KS Neufeld NH Alexopoulos GS Marino P Mulsant BH Rothschild AJ . Factor analysis of the CORE measure of psychomotor disturbance in psychotic depression: Findings from the STOP-PD II study. Psychiatry Res. (2022) 314:114648. doi: 10.1016/j.psychres.2022.114648, PMID: 35623239

[56] Koo PC Berger C Kronenberg G Bartz J Wybitul P Reis O . Combined cognitive, psychomotor and electrophysiological biomarkers in major depressive disorder. Eur Arch Psychiatry Clin Neurosci. (2019) 269:823–32. doi: 10.1007/s00406-018-0952-9, PMID: 30392042

[57] Yashar A Lamy D . Refining the dual-stage account of intertrial feature priming: Does motor response or response feature matter? Attention Perception Psychophysics. (2011) 73:2160–7. doi: 10.3758/s13414-011-0182-y, PMID: 21769533

[58] Monéger J Harika-Germaneau G Jaafari N Doolub D Warck L Selimbegovic L . Depressive self-focus bias following failure: an eye-tracking study among individuals with clinical depression. Front Psychiatry. (2024) 15:1459831. doi: 10.3389/fpsyt.2024.1459831, PMID: 39411400 PMC11473297

[59] Failing MF Theeuwes J . Exogenous visual orienting by reward. J Vision. (2014) 14:6. doi: 10.1167/14.5.6, PMID: 24819737

[60] Failing M Nissens T Pearson D Le Pelley M Theeuwes J . Oculomotor capture by stimuli that signal the availability of reward. J Neurophysiol. (2015) 114:2316–27. doi: 10.1152/jn.00441.2015, PMID: 26289464 PMC4609761

[61] Zhao X Hu J Liu M Li Q Yang Q . Immunity for counterproductive attentional capture by reward signals among individuals with depressive symptoms. Behav Res Ther. (2025) 184:104664. doi: 10.1016/j.brat.2024.104664, PMID: 39667258

[62] Zhang D Shen J Li S Gao K Gu R . I, robot: depression plays different roles in human–human and human–robot interactions. Trans Psychiatry. (2021) 11:438. doi: 10.1038/s41398-021-01567-5, PMID: 34420040 PMC8380250

[63] Solomonov N Victoria LW Lyons K Phan DK Alexopoulos GS Gunning FM . Social reward processing in depressed and healthy individuals across the lifespan: A systematic review and a preliminary coordinate-based meta-analysis of fMRI studies. Behav Brain Res. (2023) 454:114632. doi: 10.1016/j.bbr.2023.114632, PMID: 37598904 PMC10557626

[64] Gaillard C Guillod M Ernst M Federspiel A Schoebi D Recabarren RE . Striatal reactivity to reward under threat-of-shock and working memory load in adults at increased familial risk for major depression: A preliminary study. NeuroImage: Clin. (2020) 26:102193. doi: 10.1016/j.nicl.2020.102193, PMID: 32036303 PMC7011085

[65] Rappaport BI Kandala S Luby JL Barch DM . Brain reward system dysfunction in adolescence: Current, cumulative, and developmental periods of depression. Am J Psychiatry. (2020) 177:754–63. doi: 10.1176/appi.ajp.2019.19030281, PMID: 32252540 PMC12409776

[66] Becker A Gerchen MF Kirsch M Ubl B Subramaniapillai S Diener C . Frontostriatal connectivity during reward anticipation. Z für Psychol. (2017) 225:2151–604. doi: 10.1027/2151-2604/a000307

[67] Bakic J Pourtois G Jepma M Duprat R De Raedt R Baeken C . Spared internal but impaired external reward prediction error signals in major depressive disorder during reinforcement learning. Depression Anxiety. (2017) 34:89–96. doi: 10.1002/da.22576, PMID: 27781362

[68] Geugies H Mocking RJ Figueroa CA Groot PF Marsman JBC Servaas MN . Impaired reward-related learning signals in remitted unmedicated patients with recurrent depression. Brain. (2019) 142:2510–22. doi: 10.1093/brain/awz167, PMID: 31280309 PMC6734943

[69] Zhang Q Bao C Yan R Hua L Xiong T Zou H . Aberrant social reward dynamics in individuals with melancholic major depressive disorder: An ERP study. J Affect Disord. (2024) 361:751–9. doi: 10.1016/j.jad.2024.06.043, PMID: 38885845

[70] Stemmann H Freiwald WA . Evidence for an attentional priority map in inferotemporal cortex. Proc Natl Acad Sci. (2019) 116:23797–805. doi: 10.1073/pnas.1821866116, PMID: 31685625 PMC6876153

[71] Bottini S . Social reward processing in individuals with autism spectrum disorder: A systematic review of the social motivation hypothesis. Res Autism Spectr Disord. (2018) 45:9–26. doi: 10.1016/j.rasd.2017.10.001

[72] Morris SL Call NA Mevers JL Taylor PM . A behavioral economic measure of sensitivity to social rewards in children with and without autism spectrum disorder. Res In Autism. (2025) 127:202663. doi: 10.1016/j.reia.2025.202663 PMC13521615 42663006

[73] Chiappini E Massaccesi C Korb S Steyrl D Willeit M Silani G . Neural hyperresponsivity during the anticipation of tangible social and nonsocial rewards in autism spectrum disorder: A concurrent neuroimaging and facial electromyography study. Biol Psychiatry: Cogn Neurosci Neuroimaging. (2024) 9:948–57. doi: 10.1016/j.bpsc.2024.04.006, PMID: 38642898

[74] Dubey I Ropar D de C Hamilton AF . Measuring the value of social engagement in adults with and without autism. Mol Autism. (2015) 6:35. doi: 10.1186/s13229-015-0031-2, PMID: 26097674 PMC4473830

[75] Baumeister S Moessnang C Bast N Hohmann S Aggensteiner P Kaiser A . Processing of social and monetary rewards in autism spectrum disorders. Br J Psychiatry. (2023) 222:100–11. doi: 10.1192/bjp.2022.157, PMID: 36700346 PMC9929925

[76] Choi U Kim S Sim HJ Lee S Park S Jeong JA . Abnormal brain activity in social reward learning in children with autism spectrum disorder: An fmri study. Yonsei Med J. (2015) 56:705–11. doi: 10.3349/ymj.2015.56.3.705, PMID: 25837176 PMC4397440

[77] Lin A Rangel A Adolphs R . Impaired learning of social compared to monetary rewards in autism. Front Neurosci. (2012) 6:143. doi: 10.3389/fnins.2012.00143, PMID: 23060743 PMC3461406

[78] Sequeira SL Silk JS Jones NP Forbes EE Hanson JL Hallion LS . Pathways to adolescent social anxiety: Testing interactions between neural social reward function and perceived social threat in daily life. Dev Psychopathol. (2025) 37:1214–29. doi: 10.1017/S0954579424001068, PMID: 38801123 PMC11599470

[79] Flett GL Vredenburg K Krames L . The continuity of depression in clinical and nonclinical samples. psychol Bull. (1997) 121:395–416. doi: 10.1037/0033-2909.121.3.395, PMID: 9136642

[80] Failing MF Theeuwes J . Nonspatial attentional capture by previously rewarded scene semantics. Visual Cogn. (2015) 23:82–104. doi: 10.1080/13506285.2014.990546

[81] Kilford EJ Foulkes L Blakemore SJ . Associations between age, social reward processing and social anxiety symptoms. Curr Psychol (New Brunswick N.J.). (2023) 43:1–18. doi: 10.1007/s12144-023-04551-y, PMID: 37359660 PMC10113964

